# Measures to assess commonly experienced symptoms for people with dementia in long-term care settings: a systematic review

**DOI:** 10.1186/s12916-016-0582-x

**Published:** 2016-02-26

**Authors:** Clare Ellis-Smith, Catherine J. Evans, Anna E. Bone, Lesley A. Henson, Mendwas Dzingina, Pauline M. Kane, Irene J. Higginson, Barbara A. Daveson, on behalf of BuildCARE

**Affiliations:** King’s College London, Cicely Saunders Institute, Department of Palliative Care, Policy and Rehabilitation, Bessemer Road, London, SE5 9PJ UK

**Keywords:** Dementia, Long-term care, Palliative care, Review, Symptom assessment

## Abstract

**Background:**

High symptom burden is common in long-term care residents with dementia and results in distress and behavioral challenges if undetected. Physicians may have limited time to regularly examine all residents, particularly those unable to self-report, and may rely on reports from caregivers who are frequently in a good position to detect symptoms quickly. We aimed to identify proxy-completed assessment measures of symptoms experienced by people with dementia, and critically appraise the psychometric properties and applicability for use in long-term care settings by caregivers.

**Methods:**

We searched Medline, EMBASE, PsycINFO, CINAHL and ASSIA from inception to 23 June 2015, supplemented by citation and reference searches. The search strategy used a combination of terms: dementia OR long-term care AND assessment AND symptoms (e.g. pain). Studies were included if they evaluated psychometric properties of proxy-completed symptom assessment measures for people with dementia in any setting or those of mixed cognitive abilities residing in long-term care settings. Measures were included if they did not require clinical training, and used proxy-observed behaviors to support assessment in verbally compromised people with dementia. Data were extracted on study setting and sample, measurement properties and psychometric properties. Measures were independently evaluated by two investigators using quality criteria for measurement properties, and evaluated for clinical applicability in long-term settings.

**Results:**

Of the 19,942 studies identified, 40 studies evaluating 32 measures assessing pain (n = 12), oral health (n = 2), multiple neuropsychiatric symptoms (n = 2), depression (n = 8), anxiety (n = 2), psychological wellbeing (n = 4), and discomfort (n = 2) were included. The majority of studies (31/40) were conducted in long-term care settings although none of the neuropsychiatric or anxiety measures were validated in this setting. The pain assessments, PAINAD and PACSLAC had the strongest psychometric evidence. The oral health, discomfort, and three psychological wellbeing measures were validated in this setting but require further psychometric evaluation. Depression measures were poor at detecting depression in this population. All measures require further investigation into agreement, responsiveness and interpretability.

**Conclusions:**

Measures for pain are best developed for this population and setting. All other measures require further validation. A multi-symptom measure to support comprehensive assessment and monitoring in this population is required.

**Electronic supplementary material:**

The online version of this article (doi:10.1186/s12916-016-0582-x) contains supplementary material, which is available to authorized users.

## Background

People with dementia in long-term care settings commonly have high levels of comorbidity and symptom burden [1]. Multiple symptoms at all stages of the disease with varying prevalence are reported [1–9], notably pain (12–76 %) [2], dyspnea (8–80 %) [2], depression (9–32 %) [5, 7], anxiety (3–22 %) [5, 7], hallucinations (2–11 %) [5, 7], and delusions (18 %) [5]. Assessment is challenging, with declining verbal communication and cognition and absence of biological markers; with reliance on clinical examination. Untreated symptoms lead to distress and behavioral complications and compromises quality of life, resulting in challenges to clinical management [10] and staff burden [11].

Caregivers providing personal care are well placed to detect and monitor symptoms through daily contact and knowledge of residents [12], and refer to physicians for clinical examination and treatment. Routine use of measures in care supports systematic assessment and monitoring of symptoms, with increased access to treatment and improved outcomes [13]. However, there is limited evidence on their use in long-term care settings [13]. Requirements for such measures are that they are valid and reliable to ensure accurate assessment, responsive to change, clinically interpretable, brief and simple to use [14], and require minimal training [15]. Additionally, measures used by caregivers should not require a clinical qualification or expertise, and should support assessment through proxy-observed behaviors and signs for those residents unable to reliably self-report.

Caregiver assessment in long-term care settings is not well-established for all common symptoms, e.g. psychotic symptoms [16]. However, measures based on caregiver knowledge of the person with dementia without the requirement of clinical expertise, and validated in other settings, may have clinical applicability in long-term care settings and be transferable. With further validation, psychometrically robust and established assessment measures could support caregiver assessment of residents in long-term care.

This systematic review aimed to identify proxy-completed assessment measures of common symptoms experienced by people with dementia, and critically appraise the psychometric properties and applicability for use in long-term care settings by caregivers.

## Methods

This systematic review followed Preferred Reporting Items for Systematic Reviews and Meta-Analyses (PRISMA) (Additional file 1: PRISMA checklist) [17].

### Search strategy

We searched Medline, EMBASE, PsycINFO, CINAHL and ASSIA from inception to 9 April 2014 and updated on 23 June 2015. A search strategy was developed, informed by search strategies used in previous reviews, and a scoping review of common symptoms in people with dementia performed by the authors. A combination of MeSH and keyword terms were used: dementia [18] OR long-term care AND assessment AND symptoms [19] (e.g. pain, dyspnea, depression, dental pain; Additional file 2). The search was supplemented by reference and citation search of included articles using Scopus.

### Eligibility criteria

The population comprised people with dementia, or dementia subgroup analyzed separately, in any care setting, e.g. long-term care, inpatient hospital. All settings were included to identify validated measures with potential applicability to long-term care settings. To include measures with high applicability in long-term care, studies with mixed cognitively intact and cognitively impaired participants in these settings were included. Measures were included if they assessed symptoms using proxy-observed behaviors or signs in people whose verbal communication was compromised due to dementia, were validated in English, and were for use in routine care without the requirement of formal clinical training. Caregiver self-administered measures were included as they do not rely on clinicians or trained personnel to administer them, which reduces their applicability for use in care. We excluded studies of:

- Measures that required verbal responses from people with dementia
- Measures that were face-to-face or interview administered to proxies due to limited clinical applicability in routine care
- Behavioral measures that did not identify underlying causes of behavioral change, for example, measures of aggression and sleep disturbance
- Measures not primarily assessing symptoms, including those of frailty, cognition, functioning, disease progression, quality of life, and risk, and process measures, e.g. quality of communication
- Measures that required extensive training that may not be easily accessible or available to caregivers

Studies were identified for inclusion if they were in the English language and evaluated at least two psychometric properties (including one aspect of reliability and validity) of the full measure. Qualitative, review studies, theses, and conference abstracts were excluded.

### Study selection

One investigator (CES) reviewed titles and abstracts and excluded all those clearly irrelevant. Full text review was then conducted by one investigator (CES) to exclude those studies not meeting the inclusion criteria. Studies not clearly excluded were reviewed by a second reviewer (AEB) and the final inclusion of studies was agreed by discussion and consensus. Where further information was required to determine eligibility criteria, the authors were contacted. When authors were not contactable, a decision was made based on available information.

### Data extraction and assessment of quality criteria of measures

One reviewer (CES) extracted all data from each study into a standardized data extraction Excel template, and assessed the psychometric properties using quality criteria for measurement properties of health status questionnaires. Data extraction included (1) study setting, sample, and who the measure was administered by; (2) measurement properties, including method of administration, number of items, rating period, time to administer, and training required; and (3) psychometric properties, including content validity, internal consistency, criterion validity, construct validity, reproducibility (agreement and reliability), responsiveness, floor and ceiling effects, and interpretability [20]. Where applicable, psychometric properties were extracted on dementia subsamples. Evaluation of each psychometric property was based on detailed and well-established quality criteria [20], with four ratings: positive (strong psychometric properties using adequate design and method), intermediate (some but not all aspects of property is positive, or there is doubt about design and method used) [21], negative (psychometric property does not meet criteria despite adequate design and method), or no information. Details of methodological and quality criteria are detailed in Terwee et al. [20] but include, for example, requirement for formulated hypotheses with 75 % of hypotheses supported by findings for construct validity, intraclass correlation coefficient (ICC) or Cohen’s kappa ≥0.70 for reliability, ≤15 % obtaining highest or lowest possible scores for floor and ceiling effects, and sufficient sample size ≥50 for all (sub)groups. As quality rating of sensitivity and specificity are not included in the Terwee et al. [20] quality criteria, we calculated the sum of percentages misclassified, i.e. false positives and false negatives, as follows: [(1 – sensitivity) + (1 – specificity)] [22] and gave a positive rating for criterion validity of misclassification less than 50 %, i.e. better than chance [23]. A second reviewer (LAH, MD, or PMK) checked the data extraction and independently assessed the quality. The first and second reviewer resolved any disagreements by consensus. Where authors did not state which aspect of validity or reliability were being evaluated, the investigators made a judgement based on the methods used.

## Results

### Study selection

**Fig. 1:**
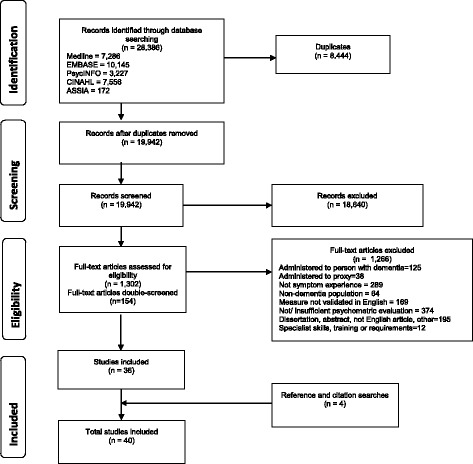
PRISMA flow chart detailing search and reasons for study exclusion

A total of 28,386 studies were identified through database searches. After deduplication, 19,942 titles and abstracts were screened, of which 1,302 were retained for full-text review. Following an independent review of 154 studies, 36 were retained for inclusion. Reasons for exclusion were required verbal responses from person with dementia (n = 125), face-to-face or interview administered to proxy (n = 38), no symptom assessment (n = 289), not dementia population (n = 64), not validated in English (n = 169), not or insufficient psychometric evaluation (n = 374), dissertation/conference abstract/study not published in English/other (n = 195), and administration required extensive training (n = 12). Following citation and reference searches, an additional four studies were identified for inclusion, resulting in a total of 40 studies (Fig. 1).

In total, 32 measures were identified, assessing pain (n = 12), oral health signs and symptoms (n = 2), multiple neuropsychiatric symptoms (n = 2), depression (n = 8), anxiety (n = 2), psychological wellbeing (n = 4), and discomfort (n = 2). The majority of studies were conducted in long-term care settings (n = 31), with seven studies recruiting from outpatient clinics [24–30]. One study was conducted in an orthopedic ward [31] and one in psychogeriatric wards [32]. Only 11 out of the 40 studies included measures administered by non-clinically trained caregivers: six pain measures [33–39], two oral health signs and symptoms measures [40, 41], two depression measures [42, 43], and one psychological wellbeing measure [44]. The neuropsychiatric symptoms [26, 30] and anxiety [24] measures were all validated with unpaid caregiver proxies in community settings. Additional file 3 provides setting and population details of each study.

Quality assessment agreement between reviewers was 86 %. Disagreements were resolved by consensus, e.g. data checking, and discussion regarding adequacy of hypotheses and whether findings supported hypotheses.

### The strength of the psychometric properties of measures validated in long-term care settings

Of those measures validated in long-term care settings, the measures with strongest psychometric properties for pain were Pain Assessment in Advanced Dementia (PAINAD) [31, 45–48], and Pain Assessment Checklist for Seniors with Limited Ability to Communicate (PACSLAC/PACSLAC-II) [35, 36, 45, 47, 49, 50]. The Oral Health Assessment Tool (OHAT) [40] and Discomfort Scale-Dementia Alzheimer’s Type (DS-DAT) [47, 51] had the strongest psychometric properties for oral health and discomfort, respectively (Table 1). The depression measures demonstrated weak abilities to accurately detect depression in this population, and all the psychological wellbeing measures require further validation. No measures achieved positive ratings for all psychometric properties with information lacking on agreement, floor and ceiling effects, responsiveness, and interpretability (Additional file 4).

**Table 1:** Evaluation of quality criteria of measures with strongest psychometric properties validated in long-term care settings

Name of measure	Content validity	Internal consistency	Criterion validity	Construct validity	Reproducibility		Responsiveness	Floor and ceiling effects	Interpretability
					Agreement	Reliability			
Pain									
PAINAD [48]	+	?	0	?	0	?	0	–	?
PAINAD [46]	?	?	0	?	0	–	0	–	0
PAINAD [47]	0	+	–	+	0	+	0	0	?
PAINAD [45]	0	?	?	+	0	+	0	0	0
PACSLAC [49]	+	?	0	?	0	0	0	0	0
PACSLAC [36]	0	0	?	?	0	?	0	0	0
PACSLAC [47]	0	?	–	+	0	+	0	0	?
PACSLAC [45]	0	?	?	+	0	+	0	0	0
PACSLAC [50]	0	0	–	?	0	+	0	0	0
PACSLAC-II [35]	+	?	0	+	0	–	0	0	0
Oral health									
OHAT [40]	+	0	?	0	0	+	?	–	0
Discomfort									
DS-DAT [51]	+	?	0	?	0	?	0	0	0
DS-DAT [47]	–	–	–	+	0	+	0	0	?

PAINAD, Pain Assessment in Advanced Dementia; PACSLAC, Pain Assessment Checklist for Seniors with Limited Ability to Communicate; OHAT, Oral Health Assessment Tool; DS-DAT, Discomfort Scale for patients with Dementia of Alzheimer’s Type

+ A positive rating indicates strong psychometric properties according to quality criteria using adequate design and method [20]

? Intermediate [21] rating indicates some but not all aspects of psychometric properties are positive, or there is doubt about the design and method used [20]

– A negative rating indicates psychometric properties do not meet criteria despite adequate design and method used [20]

0 No information provided in the paper [14]

Measures were administered through observation during provision of routine care, observations during specified activities or time periods, examinations, knowledge of resident, all available information available to caregiver, or video recordings of residents. Rating periods ranged from one minute to one month, with time taken to complete ranging from 30 seconds to 10 minutes. Measurement training ranged from none to 4 hours (where details were provided), and up to 2 days for validation purposes. Table 2 summarizes the elements of the measure (scoring, rating period), and the feasibility (measure length, time to complete, training requirements) and applicability (method of administration used in the included studies, type of training) of measures; Additional file 5 provides details of all measures.

**Table 2:** Summary of measure details, methods of administration, and feasibility and applicability in care

	Measures	Number of items (range)	Scoring (ranges)	Methods of administration	Rating period	Time to compete (range)	Training required
Pain	APS	5–60	0–5 to 0–60	Observation over specified time period	1 minute specified observation time, observation during specified activities, observations during all personal care provision	30 seconds to 5–10 minutes	Most measures do not require any formal training Two have been specifically developed for non-clinically trained care staff
	CNPI			Observation during specified activity			
	CPAT			Observation during routine care			Training for raters in the studies ranged from 5 minute training video to 2 hours, or continued training throughout data collection period
	Doloplus-2			From memory based on knowledge of resident			
	MPS						
	NOPPAIN			Ratings of video recordings of activities or pain events			
	PAINAD			Signs of pain including facial, behavioral, vocal, functional			
	PACSLAC(-II)						
	PACI						
	PADE						
	PBOICIE						
Oral health	BOHSE	8–10	0–16 to 0–20	Observation and examination	Examination period	6–8 minutes	3–4 hours of training, with calibration
	OHAT						
Neuropsychiatric	NPI-Q	12–81	0–36	Observation and knowledge of person	Last 4 weeks	5 minutes	None
	CDBQ		0–248				
Depression	BDI-modified	7–21	0–15 to 0–80	Observation, all available information sources, knowledge of person	Last week to last 4 weeks	No information	No training, 30 minutes training or provision of instructions and instruction manual
	CESD-modified						MDS coordinator usually registered nurse
	CSDD-modified/CSDD-M-LTCS						
	DDMS						
	DSS						
	CS-GDS						
	Hayes and Lohse Non-verbal Depression Scale						
	MDSDRS						
Anxiety	GAI-modified	8–20	0–20 to 8–40	Modified to be self–administered by informal caregiver, based on knowledge of person	Last week	No information	None
	PSWQ-A-modified						
Psychological wellbeing	PGCARS	5–11	0–90	Observation	5 minutes to last 24 hours	5–10 minutes	Group and one-to-one teaching sessions with supervised practice
	PWB-CIP						
	AARS						
	AER						
				Ratings of video recordings			Training for raters in the studies ranged from none to 2 days
Discomfort	DBS	9–17	0–17 to 0–102	Rated based on information from informants, observations and resident interactions	Past week	No information	MDS coordinator, usually registered nurse
	DS-DAT						
				Observation over specified period and activity program	5 minute observation or specified activity program		DS-DAT requires training and may have limited clinical applicability as complex to learn
							Training for raters included continued training throughout data collection period

APS, Abbey Pain Scale; CNPI, Checklist of Nonverbal Behaviors; CPAT, CNA Pain Assessment Tool; MPS, Mahoney Pain Assessment Tool; NOPPAIN, Non-communicative Patient’s Pain Assessment Instrument; PAINAD, Pain Assessment in Advanced Dementia; PACSLAC, Pain Assessment Checklist for Seniors with Limited Ability to Communicate; PACI, Pain Assessment in Communicatively Impaired; PADE, Pain Assessment for Dementing Elderly; PBOICIE, Pain Behaviors for Osteoarthritis Instrument for Cognitively Impaired Elders; BOHSE, Brief Oral Health Status Examination; OHAT, Oral Health Assessment Tool; NPI-Q, Neuropsychiatric Inventory Questionnaire; CDBQ, California Dementia Behavior Questionnaire; BDI-modified, Beck Depression Inventory – modified; CESD-Modified, Center for Epidemiologic Studies Depression Scale – modified; CSDD-modified, Cornell Scale for Depression in Dementia - modified; CSDD-M-LTCS, Cornell Scale for Depression in Dementia Modified for use by Long Term Care Staff; DDMS-modified, Depression in Dementia Mood Scale – modified; DSS-modified, Depression Signs Scale – modified; GDS, Geriatric Depression Scale; MDSDRS, Minimum Data Set Depression Rating Scale; GAI-modified, Geriatric Anxiety Inventory – modified; PSWQ-A-modified, Penn State Worry Questionnaire – Abbreviated – modified; PGCARS, Philadelphia Geriatric Center Affect Rating Scale; PWB-CIP, Psychological Wellbeing in Cognitively Impaired Persons; AARS, Apparent Affect Rating Scale; AER, Apparent Emotion Rating Instrument; DBS, Discomfort Behavior Scale; DS-DAT, Discomfort Scale for patients with Dementia of Alzheimer’s Type; MDS, Minimum Data Set

### Pain

The measures identified to assess pain were the Abbey Pain Scale (APS) [33, 47, 52], Checklist of Nonverbal Pain Indicators (CNPI) [45, 46, 52, 53], CNA Pain Assessment Tool [34], Doloplus-2 [52], Mahoney Pain Scale [38], Non-communicative Patient’s Pain Assessment Instrument (NOPPAIN) [45, 54], PAINAD [31, 45–48], PACSLAC [36, 45, 47, 49, 50] and PACSLAC-II [35], Pain Assessment in Communicatively Impaired [37, 50, 55], Pain Assessment for Dementing Elderly (PADE) [39, 45], and Pain Behaviors for Osteoarthritis Instrument for Cognitively Impaired Elders [29].

Of these, the PAINAD and PACSLAC have been the most extensively evaluated with the strongest psychometric properties. PAINAD has good internal consistency (Cronbach’s alpha of 0.70 and greater) [46, 47]. Inter-rater reliability is strong (kappa = 0.87 [45], ICC ≥0.87 [47]) in two studies, although one study reported an ICC of 0.24 when administered in rest situations and 0.80 during movement situations [46]. PAINAD has demonstrated good construct validity against APS, PACSLAC, CNPI, NOPPAIN, and PADE at rest and during exercise (r ≤0.62) [45, 47]. The PACSLAC demonstrated good construct validity against the NOPPAIN, CNPI, PADE, APS, and PAINAD at rest and during exercise (r ≤0.56) [45, 47]. Inter-rater reliability at rest and movement situations is consistently high (ICC ≥0.76) [45, 47, 50]. Both measures require further validation when used by caregivers as these have predominantly been evaluated when administered by trained research assistants or clinicians. PACSLAC-II is a modified and shortened version of the PACSLAC based on theoretical and evidence developments in pain assessment, and has good content validity [35]. Only one study evaluating the psychometric properties of PACSLAC-II [35] was identified, and was conducted in long-term care settings and administered by trained research assistants and caregivers. Evidence for construct validity was supported with expected strong correlations with PACSLAC, CNPI, PADE, and PAINAD in pain and non-pain conditions (r ≥0.56), and expected weak correlations with the Cornell Scale for Depression in Dementia (CSDD) (non-pain condition: r = –0.05, vaccination: r = 0.10, movement: r = –0.06). PACSLAC-II demonstrated ability to discriminate between non-pain and painful conditions (*P* <0.01). Internal consistency was strong (Cronbach’s alpha ≥0.74) and interrater reliability kappa was 0.63.

The NOPPAIN [45, 54] and CNA Pain Assessment Tool [34] are the only measures of pain developed for administration by non-clinically trained caregivers. The NOPPAIN is completed by observations carried out during routine care tasks, and is designed for easy administration with limited training [54]. NOPPAIN has high correlation (r ≤0.70) against CNPI, PACSLAC, PADE, and PAINAD with an inter-rater reliability kappa of 0.73 when administered by trained research assistants [45].

### Oral health signs and symptoms

Two measures, the Brief Oral Health Status Examination (BOHSE) [41] and the OHAT [40], were identified. Both assess oral health in long-term care residents and are administered by caregivers through oral examination of the resident. OHAT was derived from BOHSE and is simpler. OHAT comprises eight items and involves 3 hours of training to caregivers with calibration. When compared against comprehensive examination by a dentist, the Pearson correlation coefficients for each item ranged from –0.1 to 1.0 (n = 21). Test-retest reliability item-level unweighted kappa ranged from 0.51 to 0.71, with a total score ICC of 0.78. Inter-rater reliability item-level unweighted kappa ranged from 0.47 to 0.66 with a total score ICC of 0.74 (n = 485). Further testing in a larger sample is required to test validity.

### Multiple neuropsychiatric symptoms

The Neuropsychiatric Inventory Questionnaire (NPI-Q) [26] is an unpaid (usually family) caregiver self-administered version of the well-validated and extensively-used NPI [56]. The NPI is sometimes reported as a behavioral measure, but was included in this review as it assesses symptom experience, including depression, anxiety, hallucinations and delusions, and provides observational signs to support assessment of these symptoms. The NPI-Q subscales demonstrated high correlations with the original clinician-administered NPI subscales (r <0.70, n = 60). The California Dementia Behavior Questionnaire [30] was designed to assess behavior, but was included in this review as the majority of items assess the symptoms of depression and psychosis and is completed based on unpaid caregiver observations. Neither of these measures have been validated in the long-term care setting.

### Depression

Of the ten depression measures identified, two were developed for the purpose of assessment of verbally compromised people with dementia: the Minimum Data Set Depression Rating Scale [57–60], and Hayse and Lohse Non-Verbal Depression Scale [61]. The former is a seven-item scale derived from Minimum Data Set 2.0 items and developed to screen for depression by caregiver staff drawing upon observations during routine care; designed for long-term care settings it has high clinical applicability and is the most extensively psychometrically evaluated. However, evidence for detecting depression against gold-standard diagnosis of depression at a score cut-off point ≥3 is mixed with sensitivities and specificities of 0.91/0.69 (40 % misclassified, n = 82) [58] and 0.23/0.97 (80 % misclassified, n = 145) [57].

The CSDD is designed to be administered through interview with the person with dementia and a proxy but was modified for proxy-completion in two studies [32, 43]. Watson et al. [43] modified the CSDD for use by long-term caregiver staff most involved in the resident’s care using all available information to make the assessment (CSDD-M-LTCS). Modifications involved cognitive testing to remove technical language and changing response options from severity to frequency. Sensitivity and specificity against geriatric psychiatrist diagnosis was 0.33/0.86 (81 % misclassified, n = 112). Test-retest reliability was strong (≥0.70) but limited by small sample size (ICC = 0.83, n = 25) and inter-rater reliability ICC was 0.20 (n=111).

The Depression Signs Scale and Depression in Dementia Mood Scale [32] were originally designed to be administered based on clinical interview with the person with dementia and information from proxies, but were modified to be completed based on all available information to psychogeriatric ward staff.

The Geriatric Depression Scale (GDS) [27, 28, 42], Beck Depression Inventory [27], and Center for Epidemiologic Studies Depression Scale [27] were not originally developed for dementia but have been used in this population, either through clinical interview or self-report. In the included studies, they have been modified for proxy-completion by unpaid caregivers. The evidence for validity and applicability for use in long-term care by caregivers is therefore limited. One study examined caregiver-completed Collateral Source-GDS (CS-GDS) 30 and 15 versions in long-term care compared to gold standard diagnosis of depression [42]. In the dementia subsample (n = 35), sensitivities and specificities for the CS-GDS-30 and CS-GDS-15 were 0.70/0.56 (74 % misclassified) and 0.71/0.64 (66 % misclassified), respectively. Pearson correlation coefficient between CS-GDS and GDS ranged from 0.50 to 0.61 [42].

### Anxiety

Two anxiety measures, the Collateral-completed Geriatric Anxiety Inventory and the Penn-State Worry Questionnaire-Abbreviated were identified in the same study [24]. Both were modified in this study to be proxy-completed by unpaid caregivers. Sensitivity and specificity for the two measures, against gold-standard clinician-administered MINI-International Neuropsychiatric Inventory [62], were 0.62/0.93 (45 % misclassified) and 0.81/0.73 (46 % misclassified), respectively (n = 41). This study was not conducted in long-term care settings and proxies were therefore not caregiver staff. The measures’ validity and applicability in this setting were therefore not established.

### Psychological wellbeing

We identified four measures focused on psychological wellbeing. These were Psychological Wellbeing in Cognitively Impaired Persons [25], the Philadelphia Geriatric Center Affect Rating Scale (PGCARS) [63], Apparent Affect Rating Scale (AARS) [44], and the Apparent Emotion Rating Instrument (AER) [64]. The AARS and AER are both derived from PGCARS, originally developed by Lawton et al. [65]. However, this study was not included due to extensive training over 1 month provided to research assistant administrators [65].

The PGCARS, AARS, and AER were all validated in nursing home settings, and AARS and AER were administered by caregivers in the validation study. All three measure positive and negative affect, including items of pleasure, interest, anger, anxiety, and depression/sadness. All these measures require further psychometric evaluation.

### Discomfort

The term discomfort is operationalized as the presence of a negative emotional/physical state that can be observed [51]. The Discomfort Behavior Scale (DBS) was developed to assess discomfort/pain [66]; it was derived from items on the Minimum Data Set 2.0 and it has therefore been developed for use in long-term care. It is administered based on review of all available information, including direct observation and communication with residents, discussions with family, and review of records [66]. Internal consistency of DBS is positive (Cronbach alpha = 0.77, n = 9,672) [66]. However, only one psychometric evaluation of the DBS was identified and further evaluation is warranted.

The DS-DAT [47, 51] does not require clinical training. Neither of the studies identified reported the requirement for extensive training and DS-DAT was therefore included in this review. DS-DAT has, however, been critiqued as complex to use and requiring significant training [67]. As such, it may not be useful as a symptom assessment tool in routine care provision. It has demonstrated expected high correlations with pain assessment measures PAINAD, APS, and PACSLAC (≥0.63) and a strong inter-rater reliability ICC at rest (0.83) and exercise (0.85, n = 62) [47].

## Discussion

### Key findings

To our knowledge, this is the first systematic review to identify and appraise assessment measures of symptoms commonly experienced by people with dementia for use in long-term care settings. Our review identified 32 proxy-completed measures of common symptoms experienced by people with dementia. Of these measures, those that assess pain possess the strongest evidence of psychometric properties. Progress on all the other measures is promising, although oral health, psychological wellbeing, and discomfort measures require further psychometric evaluation, and there have been challenges in developing a measure that accurately detects depression. Neither of the two neuropsychiatric or two anxiety measures were validated in the long-term care setting. Furthermore, we found only 11 studies where measures were validated when administered by non-clinically trained caregivers even though these caregivers are frequently in the best position to detect changes quickly due to enhanced resident knowledge and contact [12].

Despite the extent of symptoms experienced by this population, we were unable to find any multi-symptom assessment measures validated for use in routine care as an assessment measure. Instead, we found measures that assess single symptoms or symptom groups, specifically pain, oral health signs and symptoms, multiple neuropsychiatric symptoms, depression, anxiety and psychological wellbeing, and discomfort. Assessing discomfort may alert caregivers to physical or emotional discomfort that can then be further investigated to determine the underlying cause [68]. However, content analyses of pain and discomfort measures in dementia found significant overlap resulting in poor sensitivity in assessing these constructs [69], a finding supported by our results with pain and discomfort frequently being used interchangeably. An alternative to assessing discomfort is to provide caregivers with measures to assess all common symptoms. This would facilitate a comprehensive symptom assessment, and alert caregivers to consider all common symptoms and sources of distress.

Caregivers’ use of a battery of single assessments (e.g. pain, neuropsychiatric symptoms, oral health) could facilitate detection and monitoring of common symptoms, but is unlikely to be feasible for regular and frequent use due to the time taken to complete multiple measures. Palliative or end-of-life measures, such as the Symptom Management at the End of Life in Dementia [70] or the Palliative care Outcome Scale [71] could provide a brief yet comprehensive assessment of common physical, psychological, and other distressing (such as agitation) [70] symptoms to support detection and management of symptoms in care. The former was developed to measure outcomes and evaluate end-of-life care in dementia and has been extensively evaluated [70, 72–74], although predominantly after the death of the resident. It incorporates nine symptoms in people dying with dementia and therefore has the potential for use as a clinical assessment measure for people in the dying phase. The Palliative care Outcome Scale was developed for a non-dementia population but has sound psychometric properties and is used across settings to support clinical care [75]. It has been used to assess symptoms and the quality of palliative care to nursing home patients with and without dementia [76] and found to have the potential to identify areas of care that require addressing. Nonetheless, there was a high level of missing scores for some items (≤59.8 %) in the dementia subgroup, suggesting adaptation is required for this population [76]. Results of a qualitative study suggest that such multi-symptom measures used in routine care may require provision of proxy-observed behaviors or signs to assess verbally-compromised residents with dementia [77]. Use of a single multi-symptom measure may not provide a detailed assessment of each symptom. However, multi-symptom measures may support comprehensive assessment of symptoms with minimal time burden and, if required, inform requirement for further assessment or prompt referral to health professionals.

The second major finding from our study is the lack of assessment measures to assess common symptoms. The clinical challenges and importance of accurately assessing pain in this population is apparent by substantial development in pain measures, evidenced by a recent meta-review [78]. As a consequence, we found pain measures have the strongest psychometric evidence. Nonetheless, despite the prevalence of other common symptoms in residents with dementia, such as nausea, constipation, and dyspnea, we were unable to identify any measures to assess these. With further evaluation, the Respiratory Distress Observation Scale-Family (RDOS-Family) [79] has potential to be an important measure for detecting dyspnea in long-term care residents with dementia. The original RDOS was designed for cognitively impaired adults unable to self-report but required clinical expertise to administer [80]. RDOS-Family is family caregiver self-administered based on observations with a 20-minute training provided. It has good inter-rater reliability (ICC = 0.71) between family and trained research assistants when used with patients hospitalized for conditions with dyspnea.

The stringent methodological requirements of the quality criteria and the challenges of conducting research in verbally compromised people with dementia resulted in no measures achieving positive ratings for all psychometric properties in the review. In particular, this review shows that detecting depression in people unable to self-report in this setting is challenging and that caregivers’ use of observational signs may be insufficient to assess depression. Self-report, or a clinician-administered observer-rated scale for those with moderate to severe dementia, has been recommended for assessment of depression in nursing home residents [81]. The MDS 3.0 takes this approach with the embedded Patient Health Questionnaire-9 Observational Version designed for residents unable to self-report based on observations [82]. It is completed by trained nurse assessors through interview with a caregiver who knows the resident, thus combining clinician expertise with caregiver knowledge of the resident. The Patient Health Questionnaire-9 Observational Version demonstrated strong correlation (r = 0.84, n = 48) with trained research nurse-administered CSDD [83].

We included studies conducted in all settings and some measures therefore require further validation in long-term care settings. Where measures do not exist for symptom assessment in long-term care, this review informs selection of measures for further validation by reporting strength of psychometric properties and potential applicability in long-term care settings.

This systematic review identifies and critically appraises measures of common symptoms in the dementia population in long-term care; however, there are a number of limitations. Screening measures are used to detect diagnoses such as depression and anxiety. Studies evaluating screening measures may not have been detected or met the inclusion criteria for this study. Furthermore, the quality criteria used in this review were not developed to evaluate screening tools. However, using the same quality criteria provided consistency of appraisal across the included measures. We limited the study to English language-validated measures only and to publications in English only. This means measures not developed in English, such as the Dutch Rotterdam Elderly Pain Observation Scale [84], or translated measures, such as the German [85, 86] and Chinese [87] versions of the PAINAD, and Dutch [88, 89] and Italian [90] versions of the DS-DAT, are excluded. We recognize that the conclusions are therefore limited to English language measures and therefore limited to English-speaking populations and cultures, with the majority of studies conducted in English-speaking countries, predominantly the United States. This means that the most established measures with the strongest international psychometric evidence that have been validated in multiple languages, countries, or cultures are not identified as such. Finally, decisions regarding whether measures met the inclusion criteria required judgement at times. To improve objectivity, those full-texts that did not clearly meet the exclusion criteria were second reviewed and a decision reached by consensus.

## Conclusion

Assessment measures of pain are the best developed and have the strongest evidence of psychometric properties for use by caregivers in people with dementia. All other assessment measures require further evaluation when administered by caregivers in long-term care settings. A caregiver-completed multi-symptom measure to assess the full extent of symptoms in people with dementia is urgently required so that symptoms are detected and residents are referred when medical intervention is needed.

### Availability of data and materials

The datasets supporting the conclusions of this article are included within the article and its additional files.

## Additional files

Additional file 1:
**PRISMA checklist.** (DOC 63 kb) Additional file 2:
**Full search strategy – search strategy used.** (DOCX 19 kb) Additional file 3:
**Study details for included studies – population, setting, and who the measure was administered by.** (DOCX 24 kb) Additional file 4:
**Psychometric evaluation of measures – psychometric evaluation of all measures.** (DOCX 24 kb) Additional file 5:
**Summary of measure details, methods of administration, feasibility, and applicability in care – measure details, length of measure, time taken to complete, method of administration, any training required.** (DOCX 43 kb)

## Abbreviations

AARS: Apparent Affect Rating Scale
APS: Abbey Pain Scale
AER: Apparent Emotion Rating
BOHSE: Brief Oral Health Status Examination
CNPI: Checklist of Nonverbal Pain Behaviors
CSDD: Cornell Scale for Depression in Dementia
CS-GDS: Collateral Source Geriatric Depression Scale
DBS: Discomfort Behavior Scale
DS-DAT: Discomfort Scale for patients with Dementia of Alzheimer’s Type
ICC: Intraclass correlation coefficient
NOPPAIN: Non-communicative Patient’s Pain Assessment Instrument
NPI-Q: Neuropsychiatric Inventory-Questionnaire
OHAT: Oral Health Assessment Tool
PACSLAC: Pain Assessment Checklist for Seniors with Limited Ability to Communicate
PADE: Pain Assessment for the Dementing Elderly
PAINAD: Pain Assessment in Advanced Dementia
PBOICIE: Pain Behaviors for Osteoarthritis Instrument for Cognitively Impaired Elders
PGCARS: Philadelphia Geriatric Center Affect Rating Scale
RDOS: Respiratory Distress Observation Scale
