# Supplementary material for: Measures to assess commonly experienced symptoms for people with dementia in long-term care settings: a systematic review
Source: BMC Med. 2016 Feb 26;14:38. doi: 10.1186/s12916-016-0582-x (PMC4769567; doi:10.1186/s12916-016-0582-x)
Supplement: Additional file 2: — Full search strategy – search strategy used. (DOCX 19 kb) [file 12916_2016_582_MOESM2_ESM.docx]

**Additional file 2: Full search strategy^1,2^**

1. exp Dementia/

2. Delirium/

3. Wernicke Encephalopathy/

4. Delirium, Dementia, Amnestic, Cognitive Disorders/

5. dement*.mp.

6. alzheimer*.mp.

7. (lewy* adj2 bod*).mp.

8. deliri*.mp.

9. (chronic adj2 cerebrovascular).mp.

10. ("organic brain disease" or "organic brain syndrome").mp.

11. ("normal pressure hydrocephalus" and "shunt*").mp.

12. "benign senescent forgetfulness".mp.

13. (cerebr* adj2 deteriorat*).mp.

14. (cerebral* adj2 insufficient*).mp.

15. (pick* adj2 disease).mp.

16. (creutzfeldt or jcd or cjd).mp.

17. huntington*.mp.

18. binswanger*.mp.

19. korsako*.mp.

20. Long-Term Care/

21. exp Nursing Homes/

22. Assisted Living Facilities/

23. Homes for the Aged/

24. long term care.mp.

25. nursing home*.mp.

26. care home*.mp.

27. residential care home*.mp.

28. **OR/1-27**

29. Geriatric Assessment/

30. Needs Assessment/

31. Health Impact Assessment/

32. exp Nursing Assessment/

33. Symptom Assessment/

34. exp nutrition assessment/

35. Psychometrics/

36. Pain Measurement/

37. psychomet*.mp.

38. assess* instrument.mp.

39. assess* tool.mp.

40. (observ* adj5 assess*).mp.

41. **OR/29-40**

42. Palliative Care/

43. exp Terminal Care/

44. Terminally Ill/

45. Hospice Care/

46. palliative care.mp.

47. palliative treatment.mp.

48. palliative medicine.mp.

49. terminal care.mp.

50. terminally ill.mp.

51. end-of-life care.mp.

52. hospice care.mp.

53. exp Pain/

54. exp Pain Management/

55. pain.mp.

56. exp Dyspnea/

57. dyspn?ea.mp.

58. breathless*.mp.

59. Deglutition/

60. exp Deglutition Disorders/

61. swallow*.mp.

62. (swallow* adj3 problem*).mp.

63. Constipation/

64. constipat*.mp.

65. (mouth adj3 pain*).mp.

66. Toothache/

67. (dent* adj3 pain*).mp.

68. Accidental Falls/

69. fall*.mp.

70. mobil*.mp.

71. Pressure Ulcer/

72. (pressure adj3 ulcer).mp.

73. (pressure adj3 sore).mp.

74. (skin adj3 breakdown).mp.

75. exp Psychotic Disorders/

76. psychosis.mp.

77. psychotic.mp.

78. Delusions/

79. delusion*.mp.

80. Hallucinations/

81. hallucinat*.mp.

82. Depression/

83. depress*.mp.

84. exp Depressive Disorder/

85. exp Anxiety/

86. exp Anxiety Disorders/

87. anxiety.mp.

88. anxious.mp.

89. "Quality of Life"/

90. quality of life.mp.

91. qol.mp.

92. distress.mp.

93. wellbeing.mp.

94. "Activities of Daily Living"/

95. ADL*.mp.

96. activities of daily living.mp.

97. Vomiting/

98. Nausea/

99. vomi*.mp.

100. nausea.mp.

101. emesis.mp.

102. exp Sleep/

103. (sleep adj3 disorder*).mp.

104. **OR/42-103**

105. **28 and 41 and 104**

**References**

1. Woods B, Aguirre E, Spector AE, Orrell M. Cognitive stimulation to improve cognitive functioning in people with dementia. Cochrane Database Syst Rev 2012, 2:CD005562. doi: 005510.001002/14651858.CD14005562.pub14651852.

2. Hall S, Kolliakou A, Petkova H, Froggatt K, Higginson IJ. Interventions for improving palliative care for older people living in nursing care homes (review). Cochrane Database Syst Rev 2011(3):CD007132. doi:007110.001002/14651858.CD14007132.pub14651852.
