# Supplementary material for: Measures to assess commonly experienced symptoms for people with dementia in long-term care settings: a systematic review
Source: BMC Med. 2016 Feb 26;14:38. doi: 10.1186/s12916-016-0582-x (PMC4769567; doi:10.1186/s12916-016-0582-x)
Supplement: Additional file 3: — Study details for included studies – population, setting, and who the measure was administered by. (DOCX 24 kb) [file 12916_2016_582_MOESM3_ESM.docx]

# Additional file 3: Study details of included studies

| **Lead Author and date** | **Measure** | **Setting** | **Population** | **Measure administered by** |
| --- | --- | --- | --- | --- |
| Abbey 2004 [1] | APS | Residential aged care facilities in Australia | Phase 1: 52 residents with end- or late stage dementia  Phase 2: 61 residents | Registered nurses  Enrolled nurses  Nursing assistants |
| Anderson 2003 [2] | MDSDRS | Nursing homes in US | Residents aged ≥60  Baseline: n=145  3 months: n=95  21% diagnosis of dementia | Nursing home staff |
| Bradford 2013 [3] | GAI-Collateral  PSWQ-A-Collateral | Neurology, geriatrics and psychiatry clinics and dementia care day centers in US | People aged ≥50 with diagnosis of dementia and anxiety symptoms N=41 | Collateral (usually family) |
| Burgener 2005 [4] | PWB-CIP | Dementia diagnostic centers in US | People with diagnosis of dementia within 1 year  Baseline: n=96  18 months: n=73 | Primary caregiver (usually family) |
| Burrows 2000 [5] | MDSDRS | Nursing homes in US | Residents along full spectrum of cognition and function  Total: n=108  Derivation: n=81  Validation: n=27 | Nursing home nurses |
| Cervo 2009 [6] | CPAT | Long-term care facilities in US | Residents with diagnosis of dementia, MMSE≤19, GDS≥5  N=145 | CNA direct caregivers |
| Chalmers 2005 [7] | OHAT | Residential aged care facilities in Australia | Residents  Baseline: n=534  All study phases: n=455  56.5% diagnosis of dementia | Personal care attendants, registered nurses, enrolled nurses and nurse assistants |
| Chan 2014 [8] | PACSLAC-II | Long-term care facilities in Canada | Residents with dementia  N=124 | Long-term care staff |
| Cheung 2008 [9] | PACSLAC | Specialist dementia rest homes in New Zealand | Stable residents with dementia  N=52 | Caregivers working in the rest homes |
| DeWaters 2008 [10] | PAINAD | Orthopaedic unit of metropolitan hospital in US | Patients admitted for surgical repair of hip fracture, aged ≥65, cognitively impaired and intact  Total: n=25  Cognitively intact: n=13  Cognitively impaired: n=12 | Masters’-prepared nurses working as research assistants and principal investigator |
| Elanchenny 2001 [11] | DSS  DDMS  CSDD | Acute admission and continuing care teaching hospital geriatric psychiatry wards in UK | All inpatients at the time of the study  Total: n=58  Dementia: n=43 | Experienced staff nurses |
| Ersek 2010 [12] | CNPI  PAINAD | Nursing homes in US | Residents aged ≥65 with moderate to severe pain at baseline, nonverbal or unable to provide reliable self-report  N=60 | Trained research assistants |
| **Lead Author and date** | **Measure** | **Setting** | **Population** | **Measure administered by** |
| Feldt 2000 [13] | CNPI | Hospitals in US | Hip fracture patients aged ≥65  Total: n=83  Cognitively intact: n=34  Cognitively impaired: n=49 | Gerontological nurse practitioners with Master’s degrees |
| Fuchs-Lacelle 2004 [14] | PACSLAC | Long-term care facilities in Canada | Measures completed on residents with cognitive impairments and limited ability to communicate but residents not recruited to the study | Registered nurses and registered psychiatric nurses |
| Hayes 1991 [15] | Hayes and Lohse Non-Verbal Depression Scale | Nursing home in US | Sample 1: non-verbal residents (n=30)  Sample 2: cognitively intact residents (n=30)  Sample 3: 102 residents  Sample 4: 72 residents | Registered nurses and social workers |
| Horgas 2007 [16] | NOPPAIN | Assisted living facilities, skilled nursing facilities, retirement apartments in US | Residents with range of cognitive abilities  Total sample: n=40  Cognitively intact: n=20  Cognitively impaired: n=20 | Undergraduate honors nursing students |
| Hurley 1992 [17] | DS-DAT | Long-term care units of Department of Veterans Affairs hospitals in US | Residents with dementia  Study 2:n=68  Study 3: n=82 | Study investigator  Nurses with graduate degrees |
| Kaasalainen 2003 [18] | PACI | Long-term care facility in Canada | Residents aged ≥65  Total: n=130  Cognitively intact: n=20  Mildly cognitively impaired: n=30  Moderately cognitively impaired: n=40  Extremely cognitively impaired: n=40 | Study investigator and research assistant |
| Kaasalainen 2011 [19] | PACI | Long-term care/ complex continuing care facility in Canada | Residents aged 65-94 with diagnosis of dementia and secondary pain-related diagnosis  N=14 | Four study investigators from across disciplines  Long-term care staff (special care aides, registered nurses, therapists) |
| Kaasalainen  2013 [20] | PACI  PACSLAC | Long-term care homes, providing nursing and personal care in Canada | Residents with and without dementia  Total sample: n=338  49% diagnosis of dementia | Research assistants |
| Kaufer 2000 [21] | NPI-Q | Research Center Memory Disorders and Treatment Clinics | People with probable and possible Alzheimer’s disease | Caregiver-informants (usually family) |
| **Lead Author and date** | **Measure** | **Setting** | **Population** | **Measure administered by** |
| Kayser-Jones 1995 [22] | BOHSE | Nursing home in US | Residents aged ≥50  N=100  18% no or mildly cognitively impaired, 34% moderately cognitively impaired, 48% severely cognitively impaired | Registered nurses, licensed vocational nurses, certified nursing assistants |
| Koehler 2005 [23] | MDSDRS | Nursing homes in US | Residents  Total: n=704  Cognitively intact: n=209  Cognitively impaired: n=495 | MDS assessors |
| Kolanowski 2007 [24] | PGCARS | Nursing home in US | Residents with dementia  N=31 | Trained video raters |
| Lawton 1999 [25] | AARS | Nursing home in US | Study 1: residents with dementia  Study 2 (minimal training):  Mild dementia: n=39  Severe dementia: n=40  Study 2 (enhanced training):  Mild to severe cognitive impairment: n=180 | Study 1: trained research assistants  Study 2: certified nursing assistants |
| Li 2015 [26] | CS-GDS | Nursing homes in Australia | Residents  Total: n=88  Not or mild cognitively impairment: n=42  Moderate to moderately severe cognitive impairment: n=27  Severe to very severe cognitive impairment: n=19 | Caregiver staff providing ongoing care to resident or family member visiting frequently |
| Lints-Martindale 2012 [27] | CNPI  PACSLAC  PADE  PAINAD  NOPPAIN | Long-term care facilities in Canada | Residents with diagnosis of dementia  N=124 | Trained research assistants |
| Liu 2010 [28] | PAINAD  APS  PACSLAC  DS-DAT | Nursing homes in Hong Kong | Residents aged ≥65 with diagnosed osteoarthritis  Total: n=124  Cognitively intact: n=62  Cognitively impaired: n=62 | Investigator and trained research assistants |
| Logsdon 1995 [29] | Modified GDS  Modified BDI  Modified CESD | Clinic and Alzheimer’s disease research center in US | People with dementia  N=76 | Caregivers (usually family) |
| Mahoney 2008 [30] | MPS | Nursing homes in Australia | Residents with advanced dementia  N=112 | Registered nurses and nursing assistants |
| Martin 2008 [31] | MDSDRS | Complex continuing care hospitals in Canada | Residents aged ≥65, very severe dementia excluded, without diagnosis of depression on admission  N=7,818 (26.6% diagnosis of dementia) | MDS assessors |
| Neville 2014 [32] | APS  Doloplus-2  CNPI | Residential aged care facilities in Australia | Residents with diagnosis of dementia  N=157 | Nurses |
| **Lead Author and date** | **Measure** | **Setting** | **Population** | **Measure administered by** |
| Nitcher 1993 [33] | CS-GDS | Outpatient geriatric assessment center in US | Patients evaluated at the center  N=170  Cognitively intact: n=61  Cognitively impaired: n=109 | Collateral (usually family) |
| Snyder 1998 [34] | AER | Nursing homes in US | Newly-admitted residents  N=312  No or minimal cognitive impairment: n=202  Severe cognitive impairment: n=107 | Investigators and research assistants |
| Stevenson 2006 [35] | DBS | Nursing homes in US | Moderate to severely cognitively impaired residents  N=29,120 | MDS assessor |
| Tsai 2008 [36] | PBOICIE | Study 3: senior health clinic in US | Study 2: people aged ≥60 with severe cognitive impairment and diagnosis of osteoarthritis  N=8  Study 3: cognitively intact people aged ≥60 with diagnosis of osteoarthritis | Research assistants |
| Victoroff 1997 [37] | CDBQ | Outpatient dementia diagnostic and treatment centers in US | People with cognitive complaints  Total: 258  Diagnosed dementia: 245  No dementia: 13 | Primary caregivers (usually family) |
| Villanueva 2003 [38] | PADE | Long-term care facilities (skilled nursing facilities and dementia assisted-living facility | Residents with diagnosis of dementia  Study 1: n=25  Study 2: n=40 | Caregiver staff |
| Warden 2003 [39] | PAINAD | Dementia special care unit in US | Residents with a diagnosis of dementia and inability to report pain or discomfort  N=44 | Dementia special care unit professional nurses and master’s level social work intern and clinical staff |
| Watson 2009 [40] | CSDD-M-LTCS | Residential care/ assisted living settings in US | Residents aged ≥65 with range of cognitive abilities  N=112 | Caregiver staff predominantly nursing assistants |

APS: Abbey Pain Scale, MDSDRS: Minimum Data Set Depression Rating Scale, GAI-Collateral: Geriatric Anxiety Inventory-Collateral, PSWQ-A-Collateral: Penn State Worry Questionnaire –Abbreviated-Collateral, PWB-CIP: Psychological Wellbeing in Cognitively Impaired Persons, CPAT: CNA Pain Assessment Tool, OHAT: Oral Health Assessment Tool, PACSLAC: Pain Assessment Checklist for Seniors with Limited Ability to Communicate, PAINAD: Pain Assessment in Advanced Dementia, DSS: Depressive Signs Scale, DDMS: Depression in Dementia Mood Scale, CSDD: Cornell Scale for Depression in Dementia, CNPI: Checklist of Nonverbal Pain Behaviors, NOPPAIN: Non-communicative Patient’s Pain Assessment Instrument, DS-DAT: Discomfort Scale for patients with Dementia of Alzheimer’s Type, PACI: Pain Assessment in Communicatively Impaired, NPI-Q: Neuropsychiatric Inventory-Questionnaire, BOHSE: Brief Oral Health Status Examination, PGCARS: Philadelphia Geriatric Center Affect Rating Scale, AARS: Apparent Affect Rating Scale, CS-GDS: Collateral Geriatric Depression Scale, PADE: Pain Assessment for the Dementing Elderly, BDI: Beck Depression Inventory, CESD: Center for Epidemiological Studies Depression Scale, MPS: Mahoney Pain Scale, AER: Apparent Emotion Rating, DBS: Discomfort Behavior Scale, PBOICIE: Pain Behaviors for Osteoarthritis Instrument for Cognitively Impaired Elders, CDBQ: California Dementia Behavior Questionnaire

MMSE: Mini Mental State Examination, GDS: Global Deterioration Scale

**References**

1. Abbey J, Piller N, De Bellis A, Esterman A, Parker D, Giles L, et al. The Abbey pain scale: a 1-minute numerical indicator for people with end-stage dementia. Int J Palliat Nurs 2004, 10:6-13.
2. Anderson RL, Buckwalter KC, Buchanan RJ, Maas ML, Imhof SL. Validity and reliability of the Minimun Data Set Depression Rating Scale (MDSDRS) for older adults in nursing homes. Age Ageing 2003, 32:435-438.
3. Bradford A, Brenes GA, Robinson RA, Wilson N, Snow AL, Kunik ME, et al. Concordance of self- and proxy-rated worry and anxiety symptoms in older adults with dementia. J Anxiety Disord 2013, 27:125-130.
4. Burgener SC, Twigg P, Popovich A. Measuring psychological well-being in cognitively impaired persons. Dementia 2005, 4:463-485.
5. Burrows AB, Morris JN, Simon SE, Hirdes JP, Phillips C. Development of a Minimum Data Set-based depression rating scale for use in nursing homes. Age Ageing 2000, 29:165-172.
6. Cervo FA, Bruckenthal P, Chen JJ, Bright-Long LE, Fields S, Zhang G. Pain assessment in nursing home residents with dementia: psychometric properties and clinical utility of the CNA Pain Assessment Tool (CPAT). J Am Med Dir Assoc 2009, 10:505-510.
7. Chalmers JM, King PL, Spencer AJ, Wright FAC, Carter KD. The oral health assessment tool--validity and reliability. Aust Dent J 2005, 50:191-199.
8. Chan S, Hadjistavropoulos T, Williams J, Lints-Martindale A. Evidence-based development and initial validation of the pain assessment checklist for seniors with limited ability to communicate-II (PACSLAC-II). Clin J Pain 2014, 30:816-824.
9. Cheung G, Choi P. The use of the Pain Assessment Checklist for Seniors with Limited Ability to Communicate (PACSLAC) by caregivers in dementia care facilities. N Z Med J 2008, 121:21-29.
10. DeWaters T, Faut-Callahan M, McCann JJ, Paice JA, Fogg L, Hollinger-Smith L, et al. Comparison of self-reported pain and the PAINAD scale in hospitalized cognitively impaired and intact older adults after hip fracture surgery. Orthop Nurs 2008, 27:21-28.
11. Elanchenny N, Shah A. Evaluation of three nurse-administered depression rating scales on acute admission and continuing care geriatric psychiatry wards. Int J Methods Psychiatr Res 2001, 10:43-51.
12. Ersek M, Herr K, Neradilek MB, Buck HG, Black B. Comparing the psychometric properties of the checklist of nonverbal pain behaviors (CNPI) and the pain assessment in advanced dementia (PAIN-AD) instruments. Pain Med 2010, 11:395-404.
13. Feldt KS. The Checklist of Nonverbal Pain Indicators (CNPI). Pain Manag Nurs 2000, 1:13-21.
14. Fuchs-Lacelle S, Hadjistavropoulos T. Development and preliminary validation of the Pain Assessment Checklist for Seniors With Limited Ability to Communicate (PACSLAC). Pain Manag Nurs 2004, 5:37-49.
15. Hayes PM, Lohse D, Bernstein I. The development and testing of the Hayes and Lohse Non-Verbal Depression Scale. Clin Gerontol 1991, 10:3-13.
16. Horgas AL, Nichols AL, Schapson CA, Vietes K. Assessing pain in persons with dementia: relationships among the non-communicative patient's pain assessment instrument, self-report, and behavioral observations. Pain Manag Nurs 2007, 8:77-85.
17. Hurley AC, Volicer BJ, Hanrahan PA, Houde S, Volicer L. Assessment of discomfort in advanced Alzheimer patients. Res Nurs Health 1992, 15:369-377.
18. Kaasalainen S, Crook J. A comparison of pain-assessment tools for use with elderly long-term-care residents. Can J Nurs Res 2003, 35:58-71.
19. Kaasalainen S, Stewart N, Middleton J, Knezacek S, Hartley T, Ife C, et al. Development and evaluation of the Pain Assessment in the Communicatively Impaired (PACI) tool: part II. Int J Palliat Nurs 2011, 17:431-438.
20. Kaasalainen S, Akhtar-Danesh N, Hadjistavropoulos T, Zwakhalen S, Verreault R. A comparison between behavioral and verbal report pain assessment tools for use with residents in long term care. Pain Manag Nurs 2013, 14:e106-e114.
21. Kaufer DI, Cummings JL, Ketchel P, Smith V, MacMillan A, Shelley T, et al. Validation of the NPI-Q, a brief clinical form of the Neuropsychiatric Inventory. J Neuropsychiatry Clin Neurosci 2000, 12:233-239.
22. Kayser-Jones J, Bird WF, Paul SM, Long L, Schell ES. An instrument to assess the oral health status of nursing home residents. Gerontologist 1995, 35:814-824.
23. Koehler M, Rabinowitz T, Hirdes J, Stones M, Carpenter GI, Fries BE, et al. Measuring depression in nursing home residents with the MDS and GDS: An observational psychometric study. BMC Geriatr 2005, 5:1471-2318.
24. Kolanowski A, Hoffman L, Hofer SM. Concordance of self-report and informant assessment of emotional well-being in nursing home residents with dementia. J Gerontol 2007, 62:20-27.
25. Lawton MP, Van Haitsma K, Perkinson M, Ruckdeschel K. Observed affect and quality of life in dementia: Further affirmations and problems. J Ment Health Aging 1999, 5:69-81.
26. Li Z, Jeon YH, Low LF, Chenoweth L, O'Connor DW, Beattie E, et al. Validity of the geriatric depression scale and the collateral source version of the geriatric depression scale in nursing homes. Int Psychogeriatr 2015, 27:1495-1504.
27. Lints-Martindale AC, Hadjistavropoulos T, Lix LM, Thorpe L. A comparative investigation of observational pain assessment tools for older adults with dementia. Clin J Pain 2012, 28:226-237.
28. Liu JYW, Briggs M, Closs SJ. The psychometric qualities of four observational pain tools (OPTs) for the assessment of pain in elderly people with osteoarthritic pain. J Pain Symptom Manage 2010, 40:582-598.
29. Logsdon RG, Teri L. Depression in Alzheimer's disease patients: Caregivers as surrogate reporters. J Am Geriatr Soc 1995, 43:150-155.
30. Mahoney AEJ, Peters L. The Mahoney pain scale: Examining pain and agitation in advanced dementia. Am J Alzheimers Dis Other Demen 2008, 23:250-261.
31. Martin L, Poss JW, Hirdes JP, Jones RN, Stones MJ, Fries BE. Predictors of a new depression diagnosis among older adults admitted to complex continuing care: Implications for the depression rating scale (DRS). Age Ageing 2008, 37:51-56.
32. Neville C, Ostini R. A psychometric evaluation of three pain rating scales for people with moderate to severe dementia. Pain Manag Nurs 2014, 15:798-806.
33. Nitcher RL, Burke WJ, Roccaforte WH, Wengel SP. A collateral source version of the Geriatric Depression Rating Scale. Am J Geriatr Psychiatry 1993, 1:143-152.
34. Snyder M, Ryden MB, Shaver P, Wang J, Savik K, Gross CR, et al. The Apparent Emotion Rating Instrument: assessing affect in cognitively impaired elders. Clin Gerontol 1998, 18:17-29.
35. Stevenson KM, Brown RL, Dahl JL, Ward SE, Brown MS. The discomfort behavior scale: a measure of discomfort in the cognitively impaired based on the minimum data set 2.0. Res Nurs Health 2006, 29:576-587.
36. Tsai PF, Beck C, Richards KC, Phillips L, Roberson PK, Evans J. The Pain Behaviors for Osteoarthritis Instrument for Cognitively Impaired Elders (PBOICIE). Res Gerontol Nurs 2008, 1:116-122.
37. Victoroff J, Nielson K, Mungas D. Caregiver and clinician assessment of behavioral disturbances: the California Dementia Behavior Questionnaire. Int Psychogeriatr 1997, 9:155-174.
38. Villanueva MR, Smith TL, Erickson JS, Lee AC, Singer CM. Pain Assessment for the Dementing Elderly (PADE): reliability and validity of a new measure. J Am Med Dir Assoc 2003, 4:1-8.
39. Warden V, Hurley AC, Volicer L. Development and Psychometric Evaluation of the Pain Assessment in Advanced Dementia (PAINAD) Scale. J Am Med Dir Assoc 2003, 4:9-15.
40. Watson LC, Zimmerman S, Cohen LW, Dominik R. Practical depression screening in residential care/assisted living: five methods compared with gold standard diagnoses. Am J Geriatr Psychiatry 2009, 17:556-564.
