# Supplementary material for: Measures to assess commonly experienced symptoms for people with dementia in long-term care settings: a systematic review
Source: BMC Med. 2016 Feb 26;14:38. doi: 10.1186/s12916-016-0582-x (PMC4769567; doi:10.1186/s12916-016-0582-x)
Supplement: Additional file 4: — Psychometric evaluation of measures – psychometric evaluation of all measures. (DOCX 24 kb) [file 12916_2016_582_MOESM4_ESM.docx]

# Additional file 4: Psychometric evaluation of measures

| **Name of measures** | **Content validity** | **Internal consistency** | **Criterion validity** | **Construct validity** | **Reproducibility** | | **Responsiveness** | **Floor and ceiling effects** | **Interpretability** |
| --- | --- | --- | --- | --- | --- | --- | --- | --- | --- |
|  |  |  |  |  | **Agreement** | **Reliability** |  |  |  |
| **Pain** | | | | | | | | | |
| APS [1] | + | ? | ? | 0 | 0 | ? | ? | 0 | 0 |
| APS [2] | 0 | + | - | + | 0 | + | 0 | 0 | ? |
| APS [3] | 0 | + | 0 | ? | 0 | + | 0 | 0 | 0 |
| CNPI [4] | ? | ? | ? | ? | 0 | ? | 0 | ? | 0 |
| CNPI [5] | 0 | ? | 0 | ? | 0 | - | 0 | - | 0 |
| CNPI [6] | 0 | ? | ? | + | 0 | + | 0 | 0 | 0 |
| CNPI [3] | 0 | + | 0 | ? | 0 | - | 0 | 0 | 0 |
| CPAT [7] | + | ? | - | ? | 0 | ? | 0 | 0 | 0 |
| Doloplus-2 [3] | 0 | + | 0 | ? | 0 | + | 0 | 0 | 0 |
| MPS [8] | + | ? | 0 | ? | 0 | ? | 0 | 0 | 0 |
| NOPPAIN [9] | + | ? | ? | ? | 0 | ? | 0 | 0 | 0 |
| NOPPAIN [6] | 0 | ? | ? | + | 0 | + | 0 | 0 | 0 |
| PAINAD [10] | + | ? | 0 | ? | 0 | ? | 0 | - | ? |
| PAINAD [11] | 0 | ? | 0 | ? | 0 | ? | 0 | ? | 0 |
| PAINAD [5] | ? | ? | 0 | ? | 0 | - | 0 | - | 0 |
| PAINAD [2] | 0 | + | - | + | 0 | + | 0 | 0 | ? |
| PAINAD [6] | 0 | ? | ? | + | 0 | + | 0 | 0 | 0 |
| PACSLAC [12] | + | ? | 0 | ? | 0 | 0 | 0 | 0 | 0 |
| PACSLAC [13] | 0 | 0 | ? | ? | 0 | ? | 0 | 0 | 0 |
| PACSLAC [2] | 0 | ? | - | + | 0 | + | 0 | 0 | ? |
| PACSLAC [6] | 0 | ? | ? | + | 0 | + | 0 | 0 | 0 |
| PACSLAC [14] | 0 | 0 | - | ? | 0 | + | 0 | 0 | 0 |
| PACSLAC-II [15] | + | ? | 0 | + | 0 | - | 0 | 0 | 0 |
| PACI [16] | ? | 0 | ? | 0 | 0 | ? | 0 | 0 | 0 |
| PACI [17] | + | 0 | 0 | ? | 0 | - | 0 | 0 | 0 |
| PACI [14] | 0 | 0 | - | ? | 0 | - | 0 | 0 | 0 |
| PADE [18] | + | ? | ? | ? | 0 | ? | 0 | 0 | 0 |
| PADE [6] | 0 | ? | ? | + | 0 | + | 0 | 0 | 0 |
| PBOICIE [19] | + | ? | ? | ? | 0 | ? | 0 | 0 | ? |

| **Name of measures** | **Content validity** | **Internal consistency** | **Criterion validity** | **Construct validity** | **Reproducibility** | | **Responsiveness** | **Floor and ceiling effects** | **Interpretability** |
| --- | --- | --- | --- | --- | --- | --- | --- | --- | --- |
|  |  |  |  |  | **Agreement** | **Reliability** |  |  |  |
| **Oral health signs and symptoms** | | | | | | | | | |
| BOHSE [20] | + | 0 | 0 | 0 | 0 | ? | 0 | 0 | 0 |
| OHAT [21] | + | 0 | ? | 0 | 0 | + | ? | - | 0 |
| **Neuropsychiatric symptoms** | | | | | | | | | |
| NPI-Q [22] | 0 | 0 | + | ? | 0 | ? | 0 | 0 | 0 |
| CDBQ [23] | + | ? | 0 | ? | 0 | ? | 0 | 0 | 0 |
| **Depression** | | | | | | | | | |
| BDI-modified [24] | 0 | ? | - | ? | 0 | 0 | 0 | 0 | 0 |
| CESD– modified [24] | 0 | ? | + | ? | 0 | 0 | 0 | 0 | 0 |
| CSDD – modified [25] | 0 | ? | ? | ? | 0 | ? | 0 | 0 | 0 |
| CSDD-M-LTCS [26] | + | 0 | - | 0 | 0 | - | 0 | 0 | 0 |
| DDMS [25] | 0 | ? | ? | ? | 0 | ? | 0 | 0 | 0 |
| DSS [25] | 0 | ? | ? | ? | 0 | ? | 0 | 0 | 0 |
| GDS-Collateral source [26] | 0 | 0 | - | ? | 0 | ? | 0 | 0 | 0 |
| GDS-Collateral source [27] | + | 0 | ? | 0 | 0 | ? | 0 | 0 | 0 |
| GDS-modified [24] | 0 | ? | + | ? | 0 | 0 | 0 | 0 | 0 |
| Hayes and Lohse Non-verbal Scale [29] | + | ? | 0 | ? | 0 | ? | 0 | 0 | ? |
| MDSDRS [30] | ? | ? | + | 0 | 0 | 0 | 0 | 0 | 0 |
| MDSDRS [31] | 0 | ? | - | 0 | 0 | - | 0 | - | 0 |
| MDSDRS [32] | 0 | ? | 0 | ? | 0 | 0 | 0 | - | 0 |
| MDSDRS [33] | ? | ? | ? | 0 | 0 | 0 | 0 | 0 | 0 |
| **Anxiety** | | | | | | | | | |
| GAI [34] | 0 | ? | ? | 0 | 0 | ? | 0 | 0 | 0 |
| PSWQ-A [34] | 0 | ? | ? | 0 | 0 | ? | 0 | 0 | 0 |
| **Psychological wellbeing** | | | | | | | | | |
| PGCARS [35] | 0 | 0 | 0 | ? | 0 | ? | 0 | 0 | 0 |
| **Name of measures** | **Content validity** | **Internal consistency** | **Criterion validity** | **Construct validity** | **Reproducibility** | | **Responsiveness** | **Floor and ceiling effects** | **Interpretability** |
|  |  |  |  |  | **Agreement** | **Reliability** |  |  |  |
| PWB-CIP [36] | + | ? | 0 | ? | 0 | 0 | 0 | 0 | 0 |
| AARS [37] | 0 | 0 | 0 | ? | 0 | - | 0 | 0 | 0 |
| AER [38] | **-** | ? | 0 | **-** | 0 | ? | 0 | 0 | ? |
| **Discomfort** | | | | | | | | | |
| DBS [39] | ? | + | 0 | ? | 0 | 0 | 0 | - | ? |
| DS-DAT[40] | + | ? | 0 | ? | 0 | ? | 0 | 0 | 0 |
| DS-DAT [2] | **-** | **-** | **-** | + | 0 | + | 0 | 0 | ? |

APS: Abbey Pain Scale, CNPI: Checklist of Nonverbal Behaviors, CPAT: CNA Pain Assessment Tool, MPS: Mahoney Pain Assessment Tool, NOPPAIN: Non-communicative Patient’s Pain Assessment Instrument, PAINAD: Pain Assessment in Advanced Dementia, PACSLAC: Pain Assessment Checklist for Seniors with Limited Ability to Communicate, PACI: Pain Assessment in Communicatively Impaired, PADE: Pain Assessment for Dementing Elderly, PBOICIE: Pain Behaviors for Osteoarthritis Instrument for Cognitively Impaired Elders, BOHSE: Brief Oral Health Status Examination, OHAT: Oral Health Assessment Tool, NPI-Q: Neuropsychiatric Inventory Questionnaire, CDBQ: California Dementia Behavior Questionnaire, BDI-modified: Beck Depression Inventory – modified, CESD-Modified: Center for Epidemiologic Studies Depression Scale – modified, CSDD-modified: Cornell Scale for Depression in Dementia, CSDD-M-LTCS: Cornell Scale for Depression in Dementia Modified for use by Long Term Care Staff, DDMS-modified: Depression in Dementia Mood Scale – modified, DSS-modified: Depression Signs Scale – modified, GDS: Geriatric Depression Scale, MDSDRS: Minimum Data Set Depression Rating Scale, GAI – modified: Geriatric Anxiety Inventory – modified, PSWQ-A-modified: Penn State Worry Questionnaire – Abbreviated – modified, PGCARS: Philadelphia Geriatric Center Affect Rating Scale, PWB-CIP: Psychological Wellbeing in Cognitively Impaired Persons, AARS: Apparent Affect Rating Scale, AER: Apparent Emotion Rating Instrument, DBS: Discomfort Behavior Scale, DS-DAT: Discomfort Scale for patients with Dementia of Alzheimer’s Type

+ a positive rating indicates strong psychometric properties according to quality criteria using adequate design and method

? Intermediate rating indicates some but not all aspects of psychometric property is positive, or there is doubt about design and method used

- a negative rating indicates psychometric property does not meet criteria despite adequate design and method used

0 No information provided in the paper

**References:**

1. Abbey J, Piller N, De Bellis A, Esterman A, Parker D, Giles L, et al. The Abbey pain scale: a 1-minute numerical indicator for people with end-stage dementia. Int J Palliat Nurs 2004, 10:6-13.
2. Liu JYW, Briggs M, Closs SJ. The psychometric qualities of four observational pain tools (OPTs) for the assessment of pain in elderly people with osteoarthritic pain. J Pain Symptom Manage 2010, 40:582-598.
3. Neville C, Ostini R. A psychometric evaluation of three pain rating scales for people with moderate to severe dementia. Pain Manag Nurs 2014, 15:798-806.
4. Feldt KS. The Checklist of Nonverbal Pain Indicators (CNPI). Pain Manag Nurs 2000, 1:13-21.
5. Ersek M, Herr K, Neradilek MB, Buck HG, Black B. Comparing the psychometric properties of the checklist of nonverbal pain behaviors (CNPI) and the pain assessment in advanced dementia (PAIN-AD) instruments. Pain Med 2010, 11:395-404.
6. Lints-Martindale AC, Hadjistavropoulos T, Lix LM, Thorpe L. A comparative investigation of observational pain assessment tools for older adults with dementia. Clin J Pain 2012, 28:226-237.
7. Cervo FA, Bruckenthal P, Chen JJ, Bright-Long LE, Fields S, Zhang G, et al. Pain assessment in nursing home residents with dementia: psychometric properties and clinical utility of the CNA Pain Assessment Tool (CPAT). J Am Med Dir Assoc 2009, 10:505-510.
8. Mahoney AEJ, Peters L. The Mahoney pain scale: Examining pain and agitation in advanced dementia. Am J Alzheimers Dis Other Demen 2008, 23:250-261.
9. Horgas AL, Nichols AL, Schapson CA, Vietes K. Assessing pain in persons with dementia: relationships among the non-communicative patient's pain assessment instrument, self-report, and behavioral observations. Pain Manag Nurs 2007, 8:77-85.
10. Warden V, Hurley AC, Volicer L. Development and Psychometric Evaluation of the Pain Assessment in Advanced Dementia (PAINAD) Scale. J Am Med Dir Assoc 2003, 4:9-15.
11. DeWaters T, Faut-Callahan M, McCann JJ, Paice JA, Fogg L, Hollinger-Smith L, et al. Comparison of self-reported pain and the PAINAD scale in hospitalized cognitively impaired and intact older adults after hip fracture surgery. Orthop Nurs 2008, 27:21-28.
12. Fuchs-Lacelle S, Hadjistavropoulos T. Development and preliminary validation of the Pain Assessment Checklist for Seniors With Limited Ability to Communicate (PACSLAC). Pain Manag Nurs 2004, 5:37-49.
13. Cheung G, Choi P. The use of the Pain Assessment Checklist for Seniors with Limited Ability to Communicate (PACSLAC) by caregivers in dementia care facilities. N Z Med J 2008, 121:21-29.
14. Kaasalainen S, Akhtar-Danesh N, Hadjistavropoulos T, Zwakhalen S, Verreault R. A comparison between behavioral and verbal report pain assessment tools for use with residents in long term care. Pain Manag Nurs 2013, 14:e106-e114.
15. Chan S, Hadjistavropoulos T, Williams J, Lints-Martindale A. Evidence-based development and initial validation of the pain assessment checklist for seniors with limited ability to communicate-II (PACSLAC-II). Clin J Pain 2014, 30:816-824.
16. Kaasalainen S, Crook J. A comparison of pain-assessment tools for use with elderly long-term-care residents. Can J Nurs Res 2003, 35:58-71.
17. Kaasalainen S, Stewart N, Middleton J, Knezacek S, Hartley T, Ife C, et al. Development and evaluation of the Pain Assessment in the Communicatively Impaired (PACI) tool: part II. Int J Palliat Nurs 2011, 17:431-438.
18. Villanueva MR, Smith TL, Erickson JS, Lee AC, Singer CM. Pain Assessment for the Dementing Elderly (PADE): reliability and validity of a new measure. J Am Med Dir Assoc 2003, 4:1-8.
19. Tsai PF, Beck C, Richards KC, Phillips L, Roberson PK, Evans J. The Pain Behaviors for Osteoarthritis Instrument for Cognitively Impaired Elders (PBOICIE). Res Gerontol Nurs 2008, 1:116-122.
20. Kayser-Jones J, Bird WF, Paul SM, Long L, Schell ES. An instrument to assess the oral health status of nursing home residents. Gerontologist 1995, 35:814-824.
21. Chalmers JM, King PL, Spencer AJ, Wright FAC, Carter KD. The oral health assessment tool--validity and reliability. Aust Dent J 2005, 50:191-199.
22. Kaufer DI, Cummings JL, Ketchel P, Smith V, MacMillan A, Shelley T, et al. Validation of the NPI-Q, a brief clinical form of the Neuropsychiatric Inventory. J Neuropsychiatry Clin Neurosci 2000, 12:233-239.
23. Victoroff J, Nielson K, Mungas D. Caregiver and clinician assessment of behavioral disturbances: the California Dementia Behavior Questionnaire. Int Psychogeriatr 1997, 9:155-174.
24. Logsdon RG, Teri L. Depression in Alzheimer's disease patients: Caregivers as surrogate reporters. J Am Geriatr Soc 1995, 43:150-155.
25. Elanchenny N, Shah A. Evaluation of three nurse-administered depression rating scales on acute admission and continuing care geriatric psychiatry wards. Int J Methods Psychiatr Res 2001, 10:43-51.
26. Watson LC, Zimmerman S, Cohen LW, Dominik R. Practical depression screening in residential care/assisted living: five methods compared with gold standard diagnoses. Am J Geriatr Psychiatry 2009, 17:556-564.
27. Nitcher RL, Burke WJ, Roccaforte WH, Wengel SP. A collateral source version of the Geriatric Depression Rating Scale. Am J Geriatr Psychiatry 1993, 1:143-152.
28. Li Z, Jeon YH, Low LF, Chenoweth L, O'Connor DW, Beattie E, et al. Validity of the geriatric depression scale and the collateral source version of the geriatric depression scale in nursing homes. Int Psychogeriatr 2015, 27:1495-1504.
29. Hayes PM, Lohse D, Bernstein I. The development and testing of the Hayes and Lohse Non-Verbal Depression Scale. Clin Gerontol 1991, 10:3-13.
30. Burrows AB, Morris JN, Simon SE, Hirdes JP, Phillips C. Development of a Minimum Data Set-based depression rating scale for use in nursing homes. Age Ageing 2000, 29:165-172.
31. Anderson RL, Buckwalter KC, Buchanan RJ, Maas ML, Imhof SL. Validity and reliability of the Minimun Data Set Depression Rating Scale (MDSDRS) for older adults in nursing homes. Age Ageing 2003, 32:435-438.
32. Koehler M, Rabinowitz T, Hirdes J, Stones M, Carpenter GI, Fries BE, et al. Measuring depression in nursing home residents with the MDS and GDS: An observational psychometric study. BMC Geriatr 2005, 5:1471-2318.
33. Martin L, Poss JW, Hirdes JP, Jones RN, Stones MJ, Fries BE. Predictors of a new depression diagnosis among older adults admitted to complex continuing care: Implications for the depression rating scale (DRS). Age Ageing 2008, 37:51-56.
34. Bradford A, Brenes GA, Robinson RA, Wilson N, Snow AL, Kunik ME, et al. Concordance of self- and proxy-rated worry and anxiety symptoms in older adults with dementia. J Anxiety Disord 2013, 27:125-130.
35. Kolanowski A, Hoffman L, Hofer SM. Concordance of self-report and informant assessment of emotional well-being in nursing home residents with dementia. J Gerontol 2007, 62:20-27.
36. Burgener SC, Twigg P, Popovich A. Measuring psychological well-being in cognitively impaired persons. Dementia 2005, 4:463-485.
37. Lawton MP, Van Haitsma K, Perkinson M, Ruckdeschel K. Observed affect and quality of life in dementia: Further affirmations and problems. J Ment Health Aging 1999, 5:69-81.
38. Snyder M, Ryden MB, Shaver P, Wang J, Savik K, Gross CR, et al. The Apparent Emotion Rating Instrument: assessing affect in cognitively impaired elders. Clin Gerontol 1998, 18:17-29.
39. Stevenson KM, Brown RL, Dahl JL, Ward SE, Brown MS. The discomfort behavior scale: a measure of discomfort in the cognitively impaired based on the minimum data set 2.0. Res Nurs Health 2006, 29:576-587.
40. Hurley AC, Volicer BJ, Hanrahan PA, Houde S, Volicer L. Assessment of discomfort in advanced Alzheimer patients. Res Nurs Health 1992, 15:369-377.
