# Supplementary material for: Measures to assess commonly experienced symptoms for people with dementia in long-term care settings: a systematic review
Source: BMC Med. 2016 Feb 26;14:38. doi: 10.1186/s12916-016-0582-x (PMC4769567; doi:10.1186/s12916-016-0582-x)
Supplement: Additional file 5: — Summary of measure details, methods of administration, feasibility, and applicability in care – measure details, length of measure, time taken to complete, method of administration, any training required. (DOCX 43 kb) [file 12916_2016_582_MOESM5_ESM.docx]

# Additional file 5: Summary of measure details, methods of administration, and feasibility and applicability in care

| **Name of measure^a^** | **Number of items** | **Scoring** | **Method of administration^a, b^** | **Rating period^a, b^** | **Time to administer^a^** | **Training required^a^** |
| --- | --- | --- | --- | --- | --- | --- |
| **Pain** | | | | | | |
| APS [**1**, 2, 3] | 6 items | Each item rated from 0 (absent) to 3 (severe)  Range 0-18 higher score indicating more severe pain | **Observation** [**1,** 3]  Observation at rest and observation of standardized exercise program [2] of:  Pain signs:  Vocalisation  Facial expression  Change in body language  Behavioral change  Physiological change  Physical changes | **Observation period [1**, 3]  Observation at rest (5 minutes) and observation of standardized exercise program (20-30 minutes) [2] | 1 minute [1] | None specified by measure developer  Training provided on correct use of measure, rater training continued throughout data collection period [2] |
| CNPI [3, **4**, 5, 6] | 6 items | Each item is dichotomously scored as present or absent under 2 conditions.  Range of subscores 0-6  Range of summed total score 0-12 higher score reflecting more pain signs | **Observation during rest and movement** [3, **4**]  Video recording of a resident at rest and during activity [5]  Video recording at baseline (at rest), control (swabbing), vaccination and movement-induced pain [6] of:  Pain signs:  Nonverbal vocalisations  Facial grimacing/ wincing  Bracing  Rubbing  Restlessness  Vocal complaints | **Observation period** [3, **4**]  1 minute observation at rest and series of care activities [5]  Baseline observation (3-5 minutes), swabbing, vaccination and movement-induced pain [6] | Less than 5 minutes [6] | None specified by measure developer |
| **Name of Measure^a^** | **Number of items** | **Scoring** | **Method of administration^a, b^** | **Rating period^a, b^** | **Time to administer^a^** | **Training required^a^** |
| CPAT **[7]** | 5 items | Each item dichotomously scored as present or absent  Range of summed total scores 0-5 with higher score indicating more pain signs | **Observation of pain signs:**  Facial expression  Behavior  Mood  Body language  Activity level | **Observation period of 1 minute** | **1 minute** | **45 minutes training provided to Certified Nursing Assistants** |
| Doloplus-2 [3] | 10 items | Each item rated from 0-3  Total score range 0-30 higher score indicating increased pain | Observation  Pain signs:  5 somatic reactions  2 psychomotor reactions  3 psychosocial reactions | Observation in different situations that could result in pain therefore not pain at specific moment in time | No information | Nurse participants received training from project team |
| MPS **[8]** | 8 items | Each item rated from 0-3  Range 0-24 higher scores reflect more pain signs | **Observations with light touch, or observation during rest or activity**  Pain signs:  Facial expression  Breathing  Vocalization  Body language  Agitation  Appetite/ sleep  Physical state | **Up to 5- minute observation or during personal care provision** | No information | **Participating nurses received 2 hour training on MPS and study procedures** |
| **Name of measure^a^** | **Number of items** | **Scoring** | **Method of administration^a, b^** | **Rating period^a, b^** | **Time to administer^a^** | **Training required^a^** |
| NOPPAIN [6, 9] | 9 care conditions  6 pain responses: presence and intensity  1 overall pain intensity | Each 6 pain responses rated dichotomously as present or absent  Likert scale of pain intensity for each pain response  Overall pain intensity (no pain, little pain, moderate pain, quite bad pain, very bad pain, pain is almost unbearable) | Video recording at baseline (at rest), control (swabbing), vaccination and movement-induced pain [6]    Video recording of activity-based protocol to elicit pain behaviors: participants asked to sit, stand, lie on a bed, walk in place, and transfer between activities for 1 minute intervals for a total of 10 minutes [9]  Pain signs:  Pain words  Pain noises  Pain faces  Rubbing  Bracing  Restlessness | Baseline observation (3-5 minutes), swabbing, vaccination and movement-induced pain [6]  Participants asked to sit, stand, lie on a bed, walk in place, and transfer between activities for 1 minute intervals for a total of 10 minutes [9] | Less than five minutes [6] | Designed to require minimal training  Raters completed brief standardized CD training program developed by measure developer [9] |
| **Name of measure^a^** | **Number of items** | **Scoring** | **Method of administration^a, b^** | **Rating period^a, b^** | **Time to administer^a^** | **Training required^a^** |
| PAINAD [2, 5, 6, **10**, 11] | 5 items | Each item rated from 0-2  Range 0-10 higher score reflects more severe pain | Observation at rest and observation of standardized exercise program [2]  Video recording of a resident at rest and during activity [5]  Video recording at baseline (at rest), control (swabbing), vaccination and movement-induced pain [6]  **Observations during rest, pleasant activity and caregiving activity [10]**  Observations of period of likely pain (transfer) and unlikely pain [11]  Pain signs:  Breathing  Negative vocalisation  Facial expression  Body language  Consolability | Observation at rest (5 minutes) and observation of standardized exercise program (20-30 minutes) [2]  1 minute observation at rest and series of care activities [5]  Baseline observation (3-5 minutes), swabbing, vaccination and movement-induced pain [6]  **5 minute observation for each activity type [10]**  5 minute observation for each period [11] | Less than 5 minutes [6] | Training provided on correct use of the measure, rater training continued throughout the data collection period [2]  Training session on measures and video scoring [5]  **2-hour training developed by measure developer [10]** |
| **Name of measure^a^** | **Number of items** | **Scoring** | **Method of administration^a, b^** | **Rating period^a, b^** | **Time to administer^a^** | **Training required^a^** |
| PACSLAC [2, 6, **12**, 13, 14] | 60 items | Dichotomous (present/ absent) for each item  Range 0-60 higher score reflect more pain signs | Observation at rest and observation of standardized exercise program [2]  Video recording at baseline (at rest), control (swabbing), vaccination and movement-induced pain [6]  **Completed from memory thinking about 2 pain events, non-pain distressing event, and time when a resident was calm [12]**  Observations while providing personal care [13]  Observation during period of rest and period of activity (naturally occurring pain) [14]  Pain signs:  Facial expressions  Activity/ body movement  Social/ personality/ mood indicators  Physiological indicators/ Eating and sleeping changes/ Vocal behaviors | Observation at rest (5 minutes) and observation of standardized exercise program (20-30 minutes) [2]  Baseline observation (3-5 minutes), swabbing, vaccination and movement-induced pain [6]  **Completed based on knowledge of and memory of residents [12]**  Following personal care provision [13]  Following observation of rest and activity [14] | 5 minutes [6, **12**, 13] | **None specified by measure developer [12]**  Training provided on correct use of measure, rater training continued throughout data collection period [2]  Completed by caregiver staff who were provided by 1-hour training by community psychogeriatric nurse and medical undergraduate researcher [13]  Five minute video training provided to research assistants on pain behaviors and pain interview with resident [14] |
| **Name of measure^a^** | **Number of items** | **Scoring** | **Method of administration^a, b^** | **Rating period^a, b^** | **Time to administer^a^** | **Training required^a^** |
| PACSLAC-II **[15]** | 31 items | Dichotomous (present/ absent) for each item  Range of 0-31 higher score reflecting more pain | **Video recording at baseline (at rest), control (swabbing), vaccination and movement-induced pain by trained research assistant**    **Observations while providing personal care by long-term care staff**  Pain signs:  Facial expressions  Verbalizations and vocalizations  Body movements  Changes in interpersonal interactions  Changes in activity patterns or routines  Mental status changes | **Baseline observation (3-5 minutes), swabbing, vaccination and movement-induced pain** | **Not specified** | **Instructions provided to long-term care staff** |
| PACI [14, 16, 17] | 7 items | Dichotomous yes/ no response  Range of 0-7 higher score reflects increased pain | Observation during period of rest and period of activity (naturally occurring pain) [14]  Observation movement-exacerbated painful event occurring in care [16]  Video recording of 2 potentially painful situations: physiotherapy and personal care [17]  Pain signs:  Facial expression  Vocal  Body movements | Following observation of rest and activity [14]  2 minute interval [16]  Observation of personal care and physiotherapy situations [17] | 10 seconds to rate following observation [17] | Five minute video training provided to research assistants on pain behaviors and pain interview with resident [14]  Study investigator and research assistant trained to use measure through five minute video of pain behaviors [16]  Brief training video of pain behaviors [17] |
| **Name of measure^a^** | **Number of items** | **Scoring** | **Method of administration^a, b^** | **Rating period^a, b^** | **Time to administer^a^** | **Training required^a^** |
| PADE [6, **18**] | 24 items | Items 1-12, 14, 22-24 are rated using a Likert scale (1-4)  Items 13, 15-21 are rated using a multiple choice score (1-4)  Part I: higher score reflects higher distress  Part III: greater dependence | Video recording at baseline (at rest), control (swabbing), vaccination and movement-induced pain [6]  **Observation of behaviour [18]**  Part I: Physical (observable facial expression, breathing pattern and posture), Part II: global assessment of pain  Part III: functional abilities | Baseline observation (3-5 minutes), swabbing, vaccination and movement-induced pain [6]  **5 minute observation [18]** | Less than 5 minutes [6]  **With practice: 5-10 minutes [18]** | **Completed by trained care staff with 1-hour training by investigators [18]** |
| PBOICIE **[19]** | 10 items | Each item rated with dichotomous response (yes/no) with range of 0-10 | **Person with dementia is rated during administration of activity protocol**  Pain signs:  Distorted ambulation or gesture  Audible expression of distress  Facial/ non-audible expression of distress  Changes in daily routine | **Duration of activity protocol** | **No information** | **Completed by trained RA by first author**  **Guidelines developed for coding each behaviour** |
| **Oral health signs and symptoms** | | | | | | |
| BOHSE **[20]** | 10 items | Each item has 3 descriptors  Rated on 3-point scale (0-2)  Range 0-20 higher score reflects less healthy oral health | **Examined BOHSE as a guide, sitting on bed or in chair** | **Examination period** | **Mean: 5.6 minutes (range 5-20)** | **Completed by nursing home staff of all grades. Two 2-hour in-service training provided with oral anatomy, common dental diseases, instruments, scoring, observed and supervised examination** |
| **Name of measure^a^** | **Number of items** | **Scoring** | **Method of administration^a, b^** | **Rating period^a, b^** | **Time to administer^a^** | **Training required^a^** |
| OHAT **[21]** | 8 items | Rated on 3-point scale (0-2)  Range 0-16 higher score less healthy oral health | **Observation and examination** | **Examination period** | **Mean: 7.9 minutes (range 1-30)** | **Three hour training and calibration** |
| **Neuropsychiatric symptoms** | | | | | | |
| NPI-Q **[22]** | Each of the 12 symptoms is assessed with 1 screening question | Screening question: yes/no  If yes, symptom severity is scored on 3-point scale 1 (mild) to 3 (severe), range 0-36 with higher score reflecting higher symptom burden | **Modified from original NPI from interview to 2-page self-administered questionnaire with written instructions. Anchor points are provided for symptom severity. Symptom severity only (not symptom frequency) is assessed** | **Last 4 weeks** | **5 minutes or less** | **Completed by unpaid caregivers with written instructions and anchor points. No training provided** |
| CDBQ **[23]** | 2 parts relating to person with dementia:  a) 62  b) 19 | a) Frequency from ‘never’ to ‘constantly’  b) Severity from ‘not present’ to ‘severe’ | **Completed by caregiver based on observations** | **Symptom present in last six months, if present, scored over last month** | **No information** | **Unpaid caregivers completed without training** |
| **Depression** | | | | | | |
| BDI-modified **[24]** | 21 | Multiple choice responses to each item with a maximum score of 63 higher score reflecting worse depression | **Original scale modified to be completed by caregiver proxies, modifications consisted of simple word changes** | **Last 2 weeks** | **No information** | **Unpaid caregivers completed without training** |
| CESD–modified  **[24]** | 20 | All items rated on a 4-point scale (0-3) frequency with range from 0-60 higher scores reflecting higher depression | **Original scale modified to be completed by caregiver proxies, modifications consisted of simple word changes** | **Last 2 weeks** | **No information** | **Unpaid caregivers completed without training** |
| **Name of measure^a^** | **Number of items** | **Scoring** | **Method of administration^a, b^** | **Rating period^a, b^** | **Time to administer^a^** | **Training required^a^** |
| CSDD–modified **[25]** | 19 | All items rated on a 3-point scale (0-2) with range 0-38 higher score reflects worse depression | **Original scale modified to be completed by proxy based on nursing observations, nurse-patient interactions, handovers, notes and informal discussions**  Mood-related signs:  Behavioral disturbance  Physical signs  Cyclic functions  Ideational disturbance | **Last 1-2 weeks** | **No information** | **Completed by geriatric psychiatry ward nursing staff who were provided with instruction and access to instruction manual** |
| CSDD-M-LTCS  **[26]** | 19 | CSDD-M-LTCS severity ratings were modified to frequency ratings | **CSDD-M-LTCS modified based on cognitive testing to support use by non-clinicians**  Mood-related signs:  Behavioral disturbance  Physical signs  Cyclic functions  Ideational disturbance | **Last week** | **No information** | **Instructions with a scoring algorithm. Training provided to long-term care staff (30 minutes)** |
| DDMS - modified **[25]** | 17 | All items rated on a 7-point scale (0-6) with range of 0-17 | **Original scale modified to be completed by proxy based on nursing observations, nurse-patient interactions, handovers, notes and informal discussions** | **Last 1-2 weeks** | **No information** | **Completed by geriatric psychiatry ward nursing staff who were provided with instruction and access to instruction manual** |
| **Name of measure^a^** | **Number of items** | **Scoring** | **Method of administration^a, b^** | **Rating period^a, b^** | **Time to administer^a^** | **Training required^a^** |
| DSS - modified **[25]** | 9 | 8 items rated 0-2,1 item rated 0-1  Range 0-17 | **Original scale modified to be completed by proxy based on nursing observations, nurse-patient interactions, handovers, notes and informal discussions** | **Last 1-2 weeks** | **No information** | **Completed by geriatric psychiatry ward nursing staff who were provided with instruction and access to instruction manual** |
| GDS - Collateral source/ informant version [**24, 27**, 28] | 15-item  30-item | Yes/no response  Range 0-15 or 0-30 depending on version used with higher score indicating worse depression | **Original scale modified through simple changes to wording to be self-completed by proxy based on knowledge of person [24]**  **Original scale modified to be self-completed by proxy based on knowledge of person [27,** 28] | **Last 2 weeks [24]**  **Last week [27,** 28] | **No information** | **Completed by unpaid caregiver or nursing home caregiver staff without training** |
| Hayes and Lohse Non-verbal Depression Scale **[29]** | 20 | 0 (almost never) to 4 (always)  Range 0-80 with higher scores indicating worse depression | **Observation of person with dementia** | **Last month** | **No information** | **Completed by staff who know resident well including registered nurses, social workers or any other professional staff. No details of training provided** |
| MDSDRS **[30**, 31, 32, 33] | 7 | 0 (not at all)-2 (daily or almost daily)  Range 0-14 with higher scores indicating worse depressions | **Informal discussion with resident, observe signs, discussions with staff and family, clinical record [30**, 31, 32, 33] | **Last 30 days** **[30**, 31, 32, 33] | No information | MDS-trained nurses |
| **Name of measure^a^** | **Number of items** | **Scoring** | **Method of administration^a, b^** | **Rating period^a, b^** | **Time to administer^a^** | **Training required^a^** |
| **Anxiety** | | | | | | |
| GAI-modified  **[34]** | 20 | Dichotomous (agree/disagree)  0-20 with higher score reflecting worse anxiety | **Normally administered to person with dementia, in this study modified for self-completion by proxy, based on knowledge of the person with dementia** | **Last week** | **No information** | **Completed by unpaid caregiver without training** |
| PSWQ-A-modified  **[34]** | 8 | 1-5 with total range from 8-40 higher scores reflecting worse anxiety | **Normally administered to person with dementia, in this study modified for self-completion by proxy, based on knowledge of the person with dementia** | **No information** | **No information** | **Completed by unpaid caregiver without training** |
| **Psychological wellbeing** |  |  |  |  |  |  |
| PGCARS [35] | 6 | Scored according time behaviour evidenced  Never  <16 seconds  16-59 seconds  1-5 minutes  >5 minutes | 20 minute video recording of residents over period of 12 days at times of high agitation or passivity  Administered based on observed behaviour:  Pleasure  Anger  Anxiety  Depression  Interest  Contentment | 20 minutes | No information | Research assistants and video raters provided with 2-day training |
| PWB-CIP **[36]** | 11 | Higher scores reflect higher wellbeing | **Administered based on observed behavior** | **Last 24 hours** | **5-10 minutes** | **Completed by primary unpaid caregiver without training** |
| **Name of measure^a^** | **Number of items** | **Scoring** | **Method of administration^a, b^** | **Rating period^a, b^** | **Time to administer^a^** | **Training required^a^** |
| AARS **[37]** | 5 | Scored according time behaviour evidenced  Never  <16 seconds  16-59 seconds  1-2 minutes  >2 minutes | **Administered based on observed behaviour. Descriptions of signs or indicators of each emotion are provided**  **Pleasure**  **Anger**  **Anxiety/Fear**  **Depression/Sadness**  **Interest** | **5 minute period although other time periods can be specified** | **No information** | **Training to certified nursing assistants:**  **Group and one-to-one teaching sessions with supervised practice** |
| AER **[38]** | 6 | Dichotomous (present/absence) for any affective state for which indicators are observed  15 points assigned for every positive state marked 1; and 15 points for every negative state marked 0  Total score range from 0 90 higher score reflecting positive state | **Observer-rated using verbal and no-verbal indicators. Rater observes resident and circles indicators observed in this time for each of the emotions. Prior information of the resident is not considered** | **5-10 minutes** | **No information** | **Training to research assistants: 2 hours including interpretation of indicators and supervised practice** |
| **Discomfort** |  |  |  |  |  |  |
| DBS **[39]** | 17 items | Each item rated 0-6, with range of 0-102 | **Rated as part of MDS based on medical record, observations, interactions with resident, family, care staff, physician** | **Past week** | **No information** | **MDS-trained nurses** |
| **Name of measure^a^** | **Number of items** | **Scoring** | **Method of administration^a, b^** | **Rating period^a, b^** | **Time to administer^a^** | **Training required^a^** |
| DS-DAT [2, **40**] | 9 items | Frequency, intensity, and duration of each of the 9 categories is scored  Range: 0-27 with higher score reflecting increased discomfort | **Observed in natural setting without stimuli [40]**  Observation at rest and observation of standardized exercise program [2]  Observation of 9 behavioral indicators:  Noisy breathing  Negative vocalisation  Content facial expression  Frightened facial expression  Frown  Relaxed body language  Fidgeting | **5 minute observation [40]**  Observation at rest (5 minutes) and observation of standardized exercise program (20-30 minutes) [2] | No information | Training provided on correct use of measure, rater training continued throughout data collection period [2]  **Training developed, but not detailed [40]** |

^a^Text and references in bold indicate data reported in the original validation study of the reported version of the measure

^b^Where details of methods of administration are not reported in studies, they are reported as following original administration methods

APS: Abbey Pain Scale, CNPI: Checklist of Nonverbal Behaviors, CPAT: CNA Pain Assessment Tool, MPS: Mahoney Pain Assessment Tool, NOPPAIN: Non-communicative Patient’s Pain Assessment Instrument, PAINAD: Pain Assessment in Advanced Dementia, PACSLAC: Pain Assessment Checklist for Seniors with Limited Ability to Communicate, PACI: Pain Assessment in Communicatively Impaired, PADE: Pain Assessment for Dementing Elderly, PBOICIE: Pain Behaviors for Osteoarthritis Instrument for Cognitively Impaired Elders, BOHSE: Brief Oral Health Status Examination, OHAT: Oral Health Assessment Tool, NPI-Q: Neuropsychiatric Inventory Questionnaire, CDBQ: California Dementia Behavior Questionnaire, BDI-modified: Beck Depression Inventory – modified, CESD-Modified: Center for Epidemiologic Studies Depression Scale – modified, CSDD-modified: Cornell Scale for Depression in Dementia, CSDD-M-LTCS: Cornell Scale for Depression in Dementia Modified for use by Long Term Care Staff, DDMS-modified: Depression in Dementia Mood Scale – modified, DSS-modified: Depression Signs Scale – modified, GDS: Geriatric Depression Scale, MDSDRS: Minimum Data Set Depression Rating Scale, GAI – modified: Geriatric Anxiety Inventory – modified, PSWQ-A-modified: Penn State Worry Questionnaire – Abbreviated – modified, PGCARS: Philadelphia Geriatric Center Affect Rating Scale, PWB-CIP: Psychological Wellbeing in Cognitively Impaired Persons, AARS: Apparent Affect Rating Scale, AER: Apparent Emotion Rating Instrument, DBS: Discomfort Behavior Scale, DS-DAT: Discomfort Scale for patients with Dementia of Alzheimer’s Type

CD: Compact disc

**References:**

1. Abbey J, Piller N, De Bellis A, Esterman A, Parker D, Giles L, et al. The Abbey pain scale: a 1-minute numerical indicator for people with end-stage dementia. Int J Palliat Nurs 2004, 10:6-13.
2. Liu JYW, Briggs M, Closs SJ. The psychometric qualities of four observational pain tools (OPTs) for the assessment of pain in elderly people with osteoarthritic pain. J Pain Symptom Manage 2010, 40:582-598.
3. Neville C, Ostini R. A psychometric evaluation of three pain rating scales for people with moderate to severe dementia. Pain Manag Nurs 2014, 15:798-806.
4. Feldt KS. The Checklist of Nonverbal Pain Indicators (CNPI). Pain Manag Nurs 2000, 1:13-21.
5. Ersek M, Herr K, Neradilek MB, Buck HG, Black B. Comparing the psychometric properties of the checklist of nonverbal pain behaviors (CNPI) and the pain assessment in advanced dementia (PAIN-AD) instruments. Pain Med 2010, 11:395-404.
6. Lints-Martindale AC, Hadjistavropoulos T, Lix LM, Thorpe L. A comparative investigation of observational pain assessment tools for older adults with dementia. Clin J Pain 2012, 28:226-237.
7. Cervo FA, Bruckenthal P, Chen JJ, Bright-Long LE, Fields S, Zhang G, et al. Pain assessment in nursing home residents with dementia: psychometric properties and clinical utility of the CNA Pain Assessment Tool (CPAT). J Am Med Dir Assoc 2009, 10:505-510.
8. Mahoney AEJ, Peters L. The Mahoney pain scale: Examining pain and agitation in advanced dementia. Am J Alzheimers Dis Other Demen 2008, 23:250-261.
9. Horgas AL, Nichols AL, Schapson CA, Vietes K. Assessing pain in persons with dementia: relationships among the non-communicative patient's pain assessment instrument, self-report, and behavioral observations. Pain Manag Nurs 2007, 8:77-85.
10. Warden V, Hurley AC, Volicer L. Development and Psychometric Evaluation of the Pain Assessment in Advanced Dementia (PAINAD) Scale. J Am Med Dir Assoc 2003, 4:9-15.
11. DeWaters T, Faut-Callahan M, McCann JJ, Paice JA, Fogg L, Hollinger-Smith L, et al. Comparison of self-reported pain and the PAINAD scale in hospitalized cognitively impaired and intact older adults after hip fracture surgery. Orthop Nurs 2008, 27:21-28.
12. Fuchs-Lacelle S, Hadjistavropoulos T. Development and preliminary validation of the Pain Assessment Checklist for Seniors With Limited Ability to Communicate (PACSLAC). Pain Manag Nurs 2004, 5:37-49.
13. Cheung G, Choi P. The use of the Pain Assessment Checklist for Seniors with Limited Ability to Communicate (PACSLAC) by caregivers in dementia care facilities. N Z Med J 2008, 121:21-29.
14. Kaasalainen S, Akhtar-Danesh N, Hadjistavropoulos T, Zwakhalen S, Verreault R. A comparison between behavioral and verbal report pain assessment tools for use with residents in long term care. Pain Manag Nurs 2013, 14:e106-e114.
15. Chan S, Hadjistavropoulos T, Williams J, Lints-Martindale A. Evidence-based development and initial validation of the pain assessment checklist for seniors with limited ability to communicate-II (PACSLAC-II). Clin J Pain 2014, 30:816-824.
16. Kaasalainen S, Crook J. A comparison of pain-assessment tools for use with elderly long-term-care residents. Can J Nurs Res 2003, 35:58-71.
17. Kaasalainen S, Stewart N, Middleton J, Knezacek S, Hartley T, Ife C, et al. Development and evaluation of the Pain Assessment in the Communicatively Impaired (PACI) tool: part II. Int J Palliat Nurs 2011, 17:431-438.
18. Villanueva MR, Smith TL, Erickson JS, Lee AC, Singer CM. Pain Assessment for the Dementing Elderly (PADE): reliability and validity of a new measure. J Am Med Dir Assoc 2003, 4:1-8.
19. Tsai PF, Beck C, Richards KC, Phillips L, Roberson PK, Evans J. The Pain Behaviors for Osteoarthritis Instrument for Cognitively Impaired Elders (PBOICIE). Res Gerontol Nurs 2008, 1:116-122.
20. Kayser-Jones J, Bird WF, Paul SM, Long L, Schell ES. An instrument to assess the oral health status of nursing home residents. Gerontologist 1995, 35:814-824.
21. Chalmers JM, King PL, Spencer AJ, Wright FAC, Carter KD. The oral health assessment tool--validity and reliability. Aust Dent J 2005, 50:191-199.
22. Kaufer DI, Cummings JL, Ketchel P, Smith V, MacMillan A, Shelley T, et al. Validation of the NPI-Q, a brief clinical form of the Neuropsychiatric Inventory. J Neuropsychiatry Clin Neurosci 2000, 12:233-239.
23. Victoroff J, Nielson K, Mungas D. Caregiver and clinician assessment of behavioral disturbances: the California Dementia Behavior Questionnaire. Int Psychogeriatr 1997, 9:155-174.
24. Logsdon RG, Teri L. Depression in Alzheimer's disease patients: Caregivers as surrogate reporters. J Am Geriatr Soc 1995, 43:150-155.
25. Elanchenny N, Shah A. Evaluation of three nurse-administered depression rating scales on acute admission and continuing care geriatric psychiatry wards. Int J Methods Psychiatr Res 2001, 10:43-51.
26. Watson LC, Zimmerman S, Cohen LW, Dominik R. Practical depression screening in residential care/assisted living: five methods compared with gold standard diagnoses. Am J Geriatr Psychiatry 2009, 17:556-564.
27. Nitcher RL, Burke WJ, Roccaforte WH, Wengel SP. A collateral source version of the Geriatric Depression Rating Scale. Am J Geriatr Psychiatry 1993, 1:143-152.
28. Li Z, Jeon YH, Low LF, Chenoweth L, O'Connor DW, Beattie E, et al. Validity of the geriatric depression scale and the collateral source version of the geriatric depression scale in nursing homes. Int Psychogeriatr 2015, 27:1495-1504.
29. Hayes PM, Lohse D, Bernstein I. The development and testing of the Hayes and Lohse Non-Verbal Depression Scale. Clin Gerontol 1991, 10:3-13.
30. Burrows AB, Morris JN, Simon SE, Hirdes JP, Phillips C. Development of a Minimum Data Set-based depression rating scale for use in nursing homes. Age Ageing 2000, 29:165-172.
31. Anderson RL, Buckwalter KC, Buchanan RJ, Maas ML, Imhof SL. Validity and reliability of the Minimun Data Set Depression Rating Scale (MDSDRS) for older adults in nursing homes. Age Ageing 2003, 32:435-438.
32. Koehler M, Rabinowitz T, Hirdes J, Stones M, Carpenter GI, Fries BE, et al. Measuring depression in nursing home residents with the MDS and GDS: An observational psychometric study. BMC Geriatr 2005, 5:1471-2318.
33. Martin L, Poss JW, Hirdes JP, Jones RN, Stones MJ, Fries BE. Predictors of a new depression diagnosis among older adults admitted to complex continuing care: Implications for the depression rating scale (DRS). Age Ageing 2008, 37:51-56.
34. Bradford A, Brenes GA, Robinson RA, Wilson N, Snow AL, Kunik ME, et al. Concordance of self- and proxy-rated worry and anxiety symptoms in older adults with dementia. J Anxiety Disord 2013, 27:125-130.
35. Kolanowski A, Hoffman L, Hofer SM. Concordance of self-report and informant assessment of emotional well-being in nursing home residents with dementia. J Gerontol 2007, 62:20-27.
36. Burgener SC, Twigg P, Popovich A. Measuring psychological well-being in cognitively impaired persons. Dementia 2005, 4:463-485.
37. Lawton MP, Van Haitsma K, Perkinson M, Ruckdeschel K. Observed affect and quality of life in dementia: Further affirmations and problems. J Ment Health Aging 1999, 5:69-81.
38. Snyder M, Ryden MB, Shaver P, Wang J, Savik K, Gross CR, et al. The Apparent Emotion Rating Instrument: assessing affect in cognitively impaired elders. Clin Gerontol 1998, 18:17-29.
39. Stevenson KM, Brown RL, Dahl JL, Ward SE, Brown MS. The discomfort behavior scale: a measure of discomfort in the cognitively impaired based on the minimum data set 2.0. Res Nurs Health 2006, 29:576-587.
40. Hurley AC, Volicer BJ, Hanrahan PA, Houde S, Volicer L. Assessment of discomfort in advanced Alzheimer patients. Res Nurs Health 1992, 15:369-377.
